# Multiplatform genomic profiling and magnetic resonance imaging identify mechanisms underlying intratumor heterogeneity in meningioma

**DOI:** 10.1038/s41467-020-18582-7

**Published:** 2020-09-23

**Authors:** Stephen T. Magill, Harish N. Vasudevan, Kyounghee Seo, Javier E. Villanueva-Meyer, Abrar Choudhury, S. John Liu, Melike Pekmezci, Sarah Findakly, Stephanie Hilz, Sydney Lastella, Benjamin Demaree, Steve E. Braunstein, Nancy Ann Oberheim Bush, Manish K. Aghi, Philip V. Theodosopoulos, Penny K. Sneed, Adam R. Abate, Mitchel S. Berger, Michael W. McDermott, Daniel A. Lim, Erik M. Ullian, Joseph F. Costello, David R. Raleigh

**Affiliations:** 1 Department of Neurological Surgery, University of California San Francisco, San Francisco, CA 94143 USA; 2 Department of Radiation Oncology, University of California San Francisco, San Francisco, CA 94143 USA; 3 Department of Radiology and Biomedical Imaging, University of California San Francisco, San Francisco, CA 94143 USA; 4 Department of Pathology, University of California San Francisco, San Francisco, CA 94143 USA; 5 Department of Bioengineering and Therapeutic Sciences, California Institute for Quantitative Biosciences, University of California San Francisco, San Francisco, CA 94143 USA; 6 Department of Ophthalmology, University of California San Francisco, San Francisco, CA 94143 USA

**Keywords:** CNS cancer, Gene regulatory networks, Cancer epigenetics, Tumour heterogeneity

## Abstract

Meningiomas are the most common primary intracranial tumors, but the molecular drivers of meningioma tumorigenesis are poorly understood. We hypothesized that investigating intratumor heterogeneity in meningiomas would elucidate biologic drivers and reveal new targets for molecular therapy. To test this hypothesis, here we perform multiplatform molecular profiling of 86 spatially-distinct samples from 13 human meningiomas. Our data reveal that regional alterations in chromosome structure underlie clonal transcriptomic, epigenomic, and histopathologic signatures in meningioma. Stereotactic co-registration of sample coordinates to preoperative magnetic resonance images further suggest that high apparent diffusion coefficient (ADC) distinguishes meningioma regions with proliferating cells enriched for developmental gene expression programs. To understand the function of these genes in meningioma, we develop a human cerebral organoid model of meningioma and validate the high ADC marker genes *CDH2* and *PTPRZ1* as potential targets for meningioma therapy using live imaging, single cell RNA sequencing, CRISPR interference, and pharmacology.

## Abstract (web summary)

Meningiomas are heterogeneous tumours. Here, the authors analysed genetic, epigenetic, and transcriptomic features across spatially-distinct meningioma samples to identify molecular programs underlying tumorigenesis that can be detected preoperatively using magnetic resonance imaging.

## Introduction

Meningiomas arising from the lining of the central nervous system are the most common primary intracranial tumors^1^. Meningiomas are thought to form from derivatives of the neural crest^2^, a multipotent embryonic cell population that exhibits remarkable genetic and functional diversity mediated by conserved gene regulatory networks. The World Health Organization (WHO) classifies meningiomas according to mitotic activity and other adverse histopathologic features^3^. According to WHO criteria, the majority of meningiomas are grade I and are typically well-controlled with surgery and radiotherapy^4,5^. In contrast, high grade meningiomas, which are defined as WHO grade II (atypical) and grade III (anaplastic), account for approximately one-third of cases and often recur despite optimal management^4,5^. Systemic and molecular therapies for meningioma patients remain investigational, and new treatments have been encumbered by the lack of relevant model systems and limited understanding of meningioma biology^6^.

Meningioma is a genetic disease that is common in patients with neurofibromatosis type 2, a complex autosomal disorder caused by germline heterozygous loss of function mutations in the tumor suppressor *NF2*^7^. While *NF2* mutations are also common in sporadic meningiomas^8,9^, clinically actionable somatic variants in meningiomas are rare and generally not associated with adverse outcomes^10–19^, with infrequent exceptions^20,21^. Classification of meningiomas based on DNA methylation predicts outcomes better than somatic variants or histologic grade^15,19,22,23^, but DNA methylation profiles have not elucidated biologic drivers or targets for molecular therapy to treat meningioma patients. We previously identified genomic and epigenetic mechanisms that activate a FOXM1/Wnt signaling axis and control meningioma cell proliferation in high grade tumors^19^. FOXM1 is a Forkhead box transcription factor that is required for cell proliferation^24–27^, and Forkhead box transcription factors are critical for meningeal development^28^. FOXM1 expression further distinguishes meningiomas at risk for recurrence^19^, but the molecular mechanisms driving this process are poorly understood, and transcription factors, such as FOXM1, are not tractable pharmacologic targets.

Thus, to elucidate biologic drivers and identify new targets for meningioma therapy, we investigated intratumor heterogeneity, a significant source of resistance to cancer treatment^29^. High grade meningiomas are characterized by genomic instability^12,19^, suggesting that regional alterations in chromosome structure may underlie treatment resistance. In support of this hypothesis, regional differences in genomic architecture^30–32^, cell proliferation, and the frequency of somatic variants^33,34^ exist in individual meningiomas. To understand the molecular mechanisms driving these differences, we stereotactically collected 86 spatially distinct samples from 13 human meningiomas, and analyzed intratumor heterogeneity using RNA sequencing, DNA methylation profiling, copy number variant (CNV) identification, tumor phylogeny generation, quantitative magnetic resonance (MR) imaging, and histopathology. We further derived meningioma cell lines from spatially distinct samples and interrogated meningioma tumorigenesis in three-dimensional (3D) coculture with human cerebral organoids using live imaging, single cell RNA sequencing, CRISPR interference (CRISPRi), and pharmacology. Our data reveal that regional alterations in chromosome structure underlie transcriptomic, epigenomic, and histopathologic signatures in meningioma. Moreover, we demonstrate that high apparent diffusion coefficient (ADC) on MR imaging can be used to preoperatively identify regions of meningiomas that are enriched for cell proliferation associated with developmental gene expression programs. By integrating our bioinformatic, histopathologic and radiologic findings with our human cerebral organoid model of meningioma, we identify *CDH2* and *PTPRZ1* as genes that are enriched in high ADC regions that drive meningioma tumorigenesis and represent novel targets for molecular therapy to treat meningioma patients. In sum, our data reveal spatial heterogeneity in meningioma on molecular and radiographic levels, and further demonstrate molecular correlates of imaging features underlying meningioma cell proliferation.

## Results

### Collection of spatially distinct meningioma samples

**Fig. 1:**
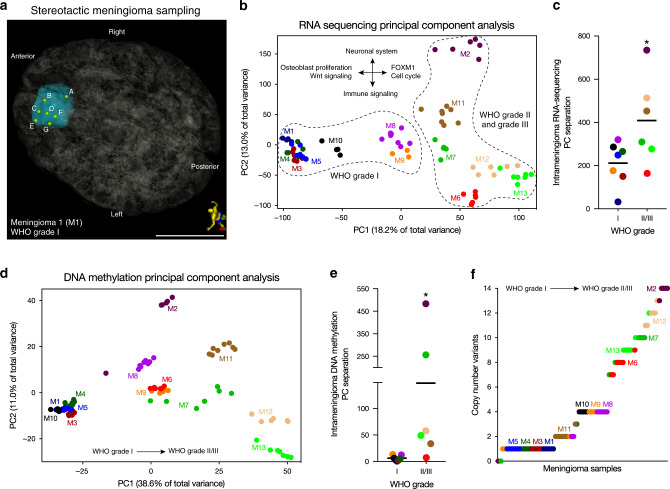
Transcriptomic, epigenomic, and genomic intratumor heterogeneity in meningioma. **a** 3D stereotactic meningioma sampling map for seven samples (**a**–**f**) from a primary WHO grade I meningioma (M1) reconstructed from preoperative MR imaging. Patient orientation is represented by the model on the bottom right. Scale bar, 5 cm. **b** RNA sequencing principal component (PC) analysis reveals that approximately 31% percent of variation among samples from the 13 meningiomas obtained for this study is explained by the first two principal components. WHO grade I meningiomas are unified by expression of genes associated with osteoblast proliferation and Wnt signaling. WHO grade II and grade III meningiomas are unified by expression of FOXM1 target genes and cell cycle effectors, and distinguished by expression of either neuronal system or immunologic genes in the second PC. **c** Intratumor RNA sequencing PC separation reveals greater heterogeneity within WHO grade II and grade III meningiomas than within WHO grade I meningiomas. **d** DNA methylation PC analysis reveals that approximately 50% of variation among samples from the meningiomas obtained for this study is explained by the first two principal components. **e** Intratumor DNA methylation PC separation confirms greater heterogeneity within WHO grades II and III meningiomas than within WHO grade I meningiomas. **f** Copy number variants derived from DNA methylation analysis reveals greater genomic instability within WHO grade II and grade III meningiomas than within WHO grade I meningiomas. Horizontal lines **c**, **f** represent means. *P ≤ 0.05, two-tailed Student’s unpaired *t* test. Samples from spatially distinct meningioma samples are color-coordinated by tumor-of-origin.

The patients included in this study were predominantly female and had a median age at diagnosis of 65 years (Supplementary Table 1). Seven meningiomas were WHO grade I (54%, 46 samples), 3 were WHO grade II (23%, 22 samples), and 3 were WHO grade III (23%, 18 samples). Seven meningiomas were sampled at the time of salvage surgery for tumor recurrence after prior treatment (54%, 48 samples), and 6 were sampled at the time of surgery for initial diagnosis (46%, 38 samples) (Supplementary Table 1). All meningiomas were located supratentorially along the midline (a potential limitation of our study in terms of generalizing our results to meningiomas in other locations) and were modeled from preoperative stereotactic MR images with the coordinates of each spatially distinct sample plotted on a 3D model (Fig. 1a). A median of 7 samples (range: 5–8) separated by a median of 14 mm (range: 2.5–177 mm) were collected from each meningioma.

### Molecular intratumor heterogeneity in meningioma

We hypothesized that spatially distinct meningioma samples would demonstrate differences in gene expression programs. To test this hypothesis, we performed RNA sequencing on 75 samples with suitable RNA, and used principal component analysis to characterize gene expression variance (Fig. 1b). Samples from each meningioma predominantly grouped together in principal component space, but there was significant intratumor heterogeneity that was most evident for high grade meningiomas (Fig. 1c). Gene ontology and chromatin enrichment analysis (ChEA)^35–37^ revealed that samples from WHO grade I meningiomas were defined by osteoblast proliferation and noncanonical Wnt gene expression programs (Fig. 1b and Supplementary Fig. 1a). In contrast, high grade meningioma samples were enriched in FOXM1 target genes along the first principal component, and were further delineated along the second principal component by expression of either neuronal or immune genes, the latter of which included increased PD-1 signaling and regulators of ectodermal development such as *CTNNB1*, *RELA*, *EED*, and *STAT6* targets (Fig. 1b and Supplementary Fig. 1a). Consistently, when we compared differentially expressed genes between WHO grade I and WHO grade III meningiomas, we found an enrichment of cell cycle, immunoregulator, and FOXM1 target genes in WHO grade III meningiomas (Supplementary Fig. 1b). The expression of *FOXM1* itself was increased in high grade meningioma samples relative to WHO grade I tumors, but there was also greater heterogeneity in *FOXM1* expression between spatially distinct samples from individual high grade meningiomas compared to grade I tumors (Supplementary Fig. 1c).

To determine if intratumor heterogeneity existed in the DNA methylation pattern of spatially distinct meningioma samples, we performed 850 K DNA methylation profiling on all 86 samples. Consistent with our transcriptomic analyses, samples from each meningioma predominantly grouped together in principal component space (Fig. 1d), but similar to our findings using RNA sequencing, there was significant intratumor heterogeneity in the DNA methylation profiles of high grade meningioma samples (Fig. 1e). Further, consistent with our previous findings^19^, high grade meningioma samples were associated with Homeobox domain gene hypermethylation and H3K27 trimethylation marks (Supplementary Fig. 2a), and had increased DNA methylation values compared to WHO grade I tumors (Supplementary Fig. 2b, c). Thus, in contrast to WHO grade I meningiomas, high grade meningiomas are characterized by significant transcriptomic and epigenomic intratumor heterogeneity.

### CNVs underlie intratumor heterogeneity in meningioma

To determine if heterogeneity existed in the CNV profile of spatially distinct meningioma samples, we identified CNVs from the DNA methylation profile on all 86 samples. The most common variant was loss of chromosome 22q in 77% of samples, which contains the tumor suppressor *NF2* (Supplementary Fig. 2d). When comparing WHO grade I and high grade meningiomas, we found an increased number of CNVs and also increased variance of CNVs per sample in high grade meningiomas (Fig. 1f).

**Fig. 2:**
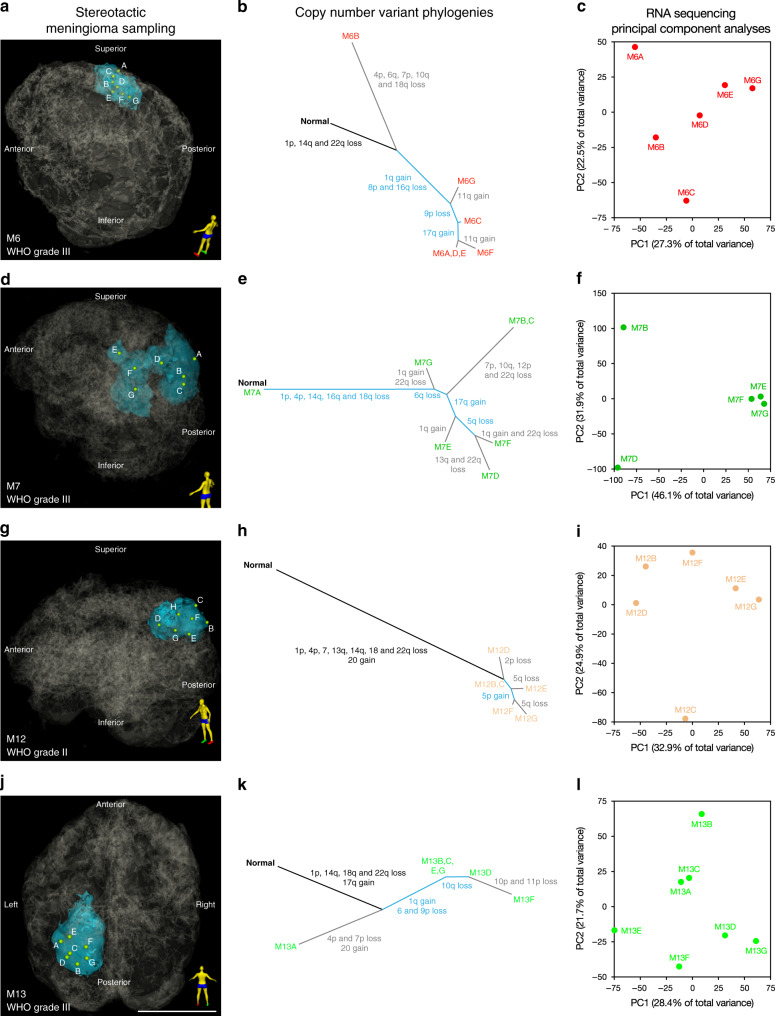
Alterations in chromosome structure underlie intratumor heterogeneity in high grade meningioma. Clonal evolution in WHO grade II and grade III meningiomas M6 **a**–**c**, M7 **d**–**f**, M12 **g**–**i**, and M13 **j**–**l**. **a, d, g, j** 3D stereotactic meningioma sampling maps reconstructed from preoperative MR imaging. Patient orientation is represented by the model on the bottom right of each panel. Scale bar, 5 cm. **b**, **e**, **h**, **k** Intratumor phylogeny based on clonal ordering of copy number variants derived from methylation analysis suggests that chromosomal structural alterations are an early event during meningioma growth. Clonal variants are shown in black, shared variants are shown in blue, and private variants alterations are shown in gray. **c**, **f**, **i**, **l** RNA sequencing principal component (PC) analysis reveals that approximately 50–78% of gene expression variation among spatially-distinct meningioma samples from individual tumors is explained by the first two principal components. Differences in immune, GPCR and hormone signaling, and mesenchymal genes delineate samples within individual meningiomas. Samples from spatially distinct meningioma samples are color-coordinated by tumor-of-origin.

To gain insights into meningioma tumorigenesis, we constructed phylogenetic trees for each meningioma based on the distribution of CNVs in spatially distinct samples (Fig. 2 and Supplementary Fig. 3a). For each tumor, 3D modeling was performed to identify sample locations in stereotactic space (Fig. 2a, d, g, and j), which were compared to CNV phylogenies (Fig. 2b, e, h, k and Supplementary Fig. 3a), RNA sequencing (Fig. 2c, f, i, and l), and DNA methylation profiles (Supplementary Fig. 3b). We found that individual WHO grade I meningiomas had uniform CNV profiles across spatially distinct samples (Supplementary Fig. 3a), but that CNV phylogenies in high grade meningiomas revealed significant intratumor heterogeneity (Fig. 2b, e, h, and k). Both WHO grade I and high grade meningiomas were characterized by clonal CNVs early in tumor evolution, suggesting that chromosomal structural alterations are an early event during meningioma tumorigenesis (Fig. 2b, e, h, k and Supplementary Fig. 3a). Although we identified CNVs affecting chromosomes 1, 14, 17, 22, and others that are recurrently altered in meningiomas^38^, there were no clear relationships between CNV identity, size, or distribution of spatially distinct samples. Nevertheless, 22q, 14q, and 1p losses were early shared events in meningioma tumor evolution, and CNV instability increased with tumor grade in a spatially distinct manner.

To determine if CNV heterogeneity in individual meningiomas was associated with epigenomic or transcriptomic differences among spatially distinct samples, we performed principal component analysis on RNA sequencing and DNA methylation profiling data from individual tumors (Fig. 2c, f, i, l, and Supplementary Fig. 3b). Gene ontology analysis among samples from individual tumors identified molecular pathways differentiating samples in high grade meningiomas, such as immune, inflammatory, and neuronal gene expression programs (Supplementary Fig. 3c). These data are consistent with our integrated analysis of all tumor samples (Fig. 1b, d), and suggest that alterations in chromosome structure underlie clonal transcriptomic and epigenomic signatures in high grade meningioma.

### Radiographic intratumor heterogeneity in meningioma

**Fig. 3:**
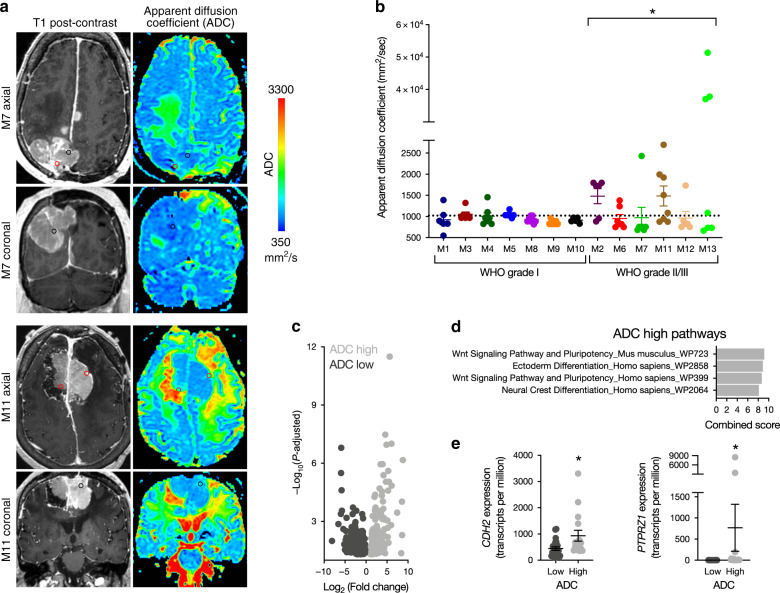
Elevated MR ADC distinguishes developmental gene expression programs in high grade meningioma. **a** Preoperative T1 post-contrast and apparent diffusion coefficient (ADC) magnetic resonance imaging of spatially distinct regions within meningiomas M7 and M11 demonstrate significant radiologic heterogeneity in high grade meningioma. Black and red circles indicate ADC low and high sample locations, respectively. **b** WHO grades II and III meningiomas have greater heterogeneity of ADC values than WHO grade I meningiomas. * denotes statistical significance between aggregate ADC values from WHO grade I and WHO grades II and III meningiomas, the latter of which were pooled for analysis. **c** RNA sequencing reveals 452 differentially expressed genes between ADC low and ADC high regions in WHO grades II and III meningiomas. **d**, **e** Gene ontology analysis from RNA sequencing demonstrates that regions with high ADC values in WHO grade II and grade III meningiomas are enriched in Wnt pathway and neuronal development genes, such as *CDH2* and *PTPRZ1*. *P ≤ 0.05, two-tailed Student’s unpaired *t* test. Mean ± standard error of the mean (as shown by error bars) are denoted for plots in (**b**, **e**). Samples from spatially distinct meningioma samples are color-coordinated by tumor-of-origin.

We hypothesized that molecular heterogeneity in meningioma could be delineated on preoperative MR imaging. To test this hypothesis, we co-registered stereotactic coordinates for all 86 spatially distinct meningioma samples with preoperative MR images, and quantified ADC and CBF at each location (Fig. 3a and Supplementary Fig. 4a). Compared to WHO grade I tumors, whole tumor ADC values were more heterogeneous among high grade meningiomas (Supplementary Fig. 4b). There also was greater ADC heterogeneity between spatially distinct samples in high grade meningioma compared to spatially distinct samples from grade I tumors (Fig. 3b), suggesting greater variability of cytoarchitectural features such as cellularity and extracellular matrix composition in WHO grade II and grade III meningiomas. CBF was also heterogeneous among spatially distinct samples, but there were no differences in the overall magnitude or heterogeneity of perfusion between samples from low and high grade meningiomas (Supplementary Fig. 4c). Thus, we performed differential gene expression analysis using our RNA sequencing data between low and high ADC samples from high grade meningiomas, dichotomized at the mean ADC value (Fig. 3c). We identified a total of 179 genes that were enriched in samples with high ADC values, and 273 genes that were enriched in samples with low ADC values (Supplementary Data 1). Gene ontology analysis revealed that high ADC samples from high grade meningiomas were enriched in Wnt, ectoderm, pluripotency, and neural crest gene expression programs (Fig. 3d), including *FOXM1* (Supplementary Fig. 4d and Supplementary Data 1). Within these gene sets, notable genes that were enriched in samples with high ADC values included *CDH2*, which encodes N-cadherin and is a regulator of noncanonical Wnt signaling and neuronal development^39,40^, and *PTPRZ1*, which is necessary for neuronal development^41^, and we and others have shown is involved in glioblastoma cell invasion and self-renewal^42–44^. Further, we previously identified high ADC as a marker of high grade meningioma and poor clinical outcomes^45,46^, suggesting that molecular correlates in regions with elevated ADC may drive meningioma tumorigenesis. In summary, these data demonstrate that radiographic intratumor heterogeneity in meningioma is associated with regional transcriptomic differences, and that high ADC distinguishes regions that are enriched in developmental gene expression programs that may underlie meningioma cell proliferation and tumor recurrence.

### Histologic intratumor heterogeneity in meningioma

**Fig. 4:**
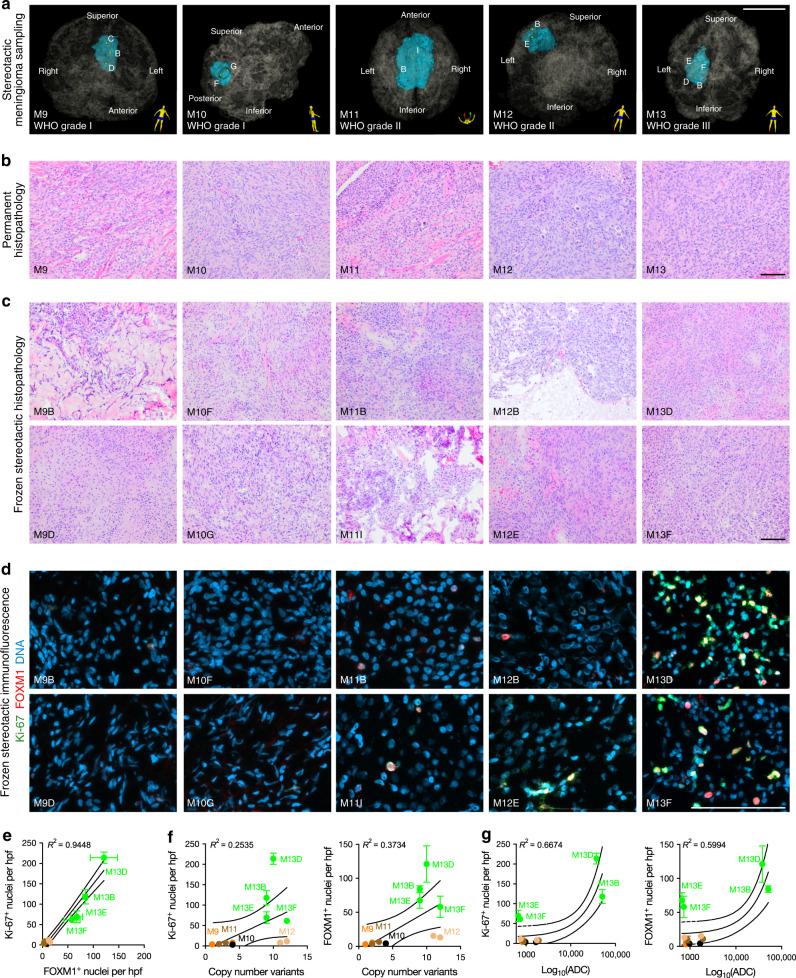
Intratumor CNVs and radiographic heterogeneity are associated with alterations in meningioma histology. **a** 3D stereotactic meningioma sampling maps reconstructed from preoperative MR imaging. Patient orientation is represented by the model on the bottom right of each panel. Scale bar, 5 cm. **b**, **c** Comparison of permanent surgical histopathology and frozen stereotactic histology from spatially distinct samples reveals intratumor and intertumor heterogeneity in meningioma. Scale bars, 100 μm. **d**, **e** Quantitative immunofluorescence for Ki-67 (green) and FOXM1 (red) demonstrates a strong positive association and elevated cell proliferation in high grade meningiomas, with heterogeneity among spatially distinct samples from individual tumors. DNA is marked with DAPI (blue). Scale bars, 100 μm. **f**, **g** Ki-67 and FOXM1 immunofluorescence demonstrate a positive association with the number of CNVs and the ADC value of each spatially distinct meningioma sample. Mean ± standard error of the mean (as shown by error bars) are denoted for each scatter plot (**e**–**g**). Samples from spatially distinct meningioma samples are color-coordinated by tumor-of-origin.

We hypothesized that intratumor histopathologic heterogeneity could be influenced by spatial differences in molecular signatures or cellular processes in meningioma, such as cell proliferation. To test this hypothesis, we performed H&E staining and immunofluorescence for FOXM1 and Ki-67, a marker of cell proliferation, on 13 spatially defined samples from 5 tumors comprising all meningioma grades (Fig. 4a and Supplementary Data 2). There was variation in the histopathology between tumors (Fig. 4b), as well as among spatially defined samples from individual meningiomas (Fig. 4c). In particular, we identified qualitative regional differences in meningioma cell size, the amount of collagen deposition, and the presence and extent of fascicles and necrosis in individual meningiomas (Fig. 4c). Notably, regional histopathologic differences were insufficient to alter the WHO grade of meningioma samples^3^. We further identified regional variation in Ki-67 labeling index and FOXM1 immunofluorescence that correlated with tumor grade, with increased heterogeneity among spatially defined WHO grade II and grade III meningioma samples (Fig. 4d, e). Ki-67 labeling index and FOXM1 immunofluorescence were positively associated with the number of CNVs per sample (Fig. 4f), and furthermore, there were positive associations between regional ADC, Ki-67 labeling index, and FOXM1 immunofluorescence (Fig. 4g). Thus, although high grade meningiomas can have globally low ADC values reflective of high cellularity (Supplementary Fig. 4b)^45^, our data suggest that regions of elevated ADC in meningiomas may harbor aggressive meningioma clones. To address the possibility that spatially-distinct samples from M13, the most clinically and histopathologically aggressive tumor in our study, may have overly biased our protein expression analyses from a limited number of samples (Fig. 4d–g), we compared *FOXM1* transcript expression to ADC values from all spatially distinct meningiomas from our study, excluding samples from M13, and validated the positive yet heterogeneous association between *FOXM1* expression and regional ADC (Supplementary Fig. 4e). Further, we found that the association between Ki-67 labeling index and FOXM1 immunofluorescence persisted in the absence of samples from M13 (Supplementary Fig. 4f), as did the positive association between FOXM1 immunofluorescence and regional CNVs (Supplementary Fig. 4g). In contrast, the associations between Ki-67 labeling index, FOXM1 immunofluorescence, and regional ADC were less strong in the absence of spatially distinct samples from M13 (Supplementary Fig. 4h). In sum, these data indicate that the MR imaging characteristics of meningiomas can be influenced by regional genomic instability and gene expression programs driving meningioma cell proliferation, but that further image-guided investigation with more patients will be required to more precisely define the spatial associations between macroscopic MR imaging features and the underlying complexity of meningioma biology.

### CDH2 and PTPRZ1 underlie meningioma formation in organoids

**Fig. 5:**
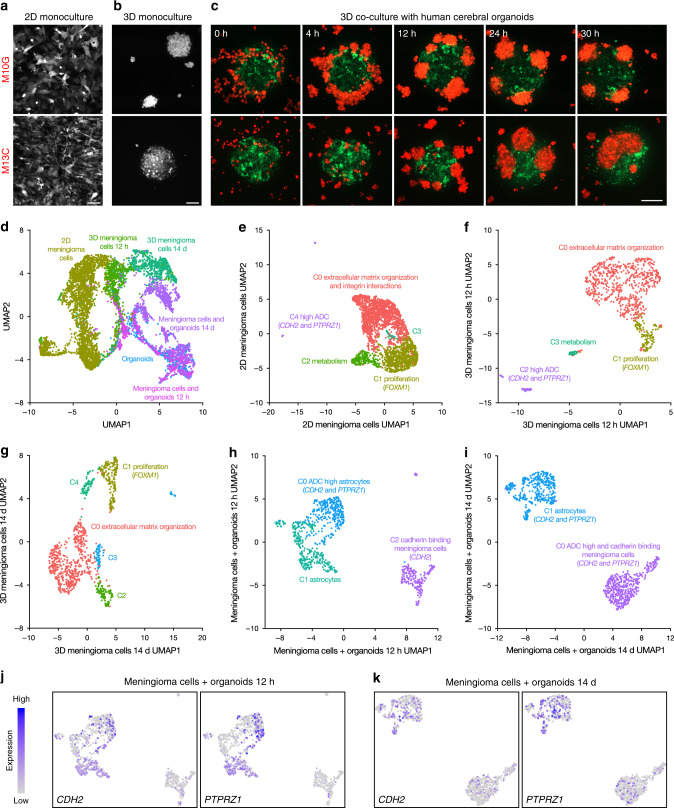
CDH2 and PTPRZ1 underlie meningioma tumorigenesis in coculture with human cerebral organoids. **a** Confocal microscopy of M10G WHO grade I and M13C WHO grade III meningioma cells labeled with red fluorescent protein in 2D culture. Scale bar, 100 μm. **b** Confocal microscopy reveals that M10G and M13C meningioma cells form tumor spheres in 3D culture. Scale bar, 100 μm. **c** Live confocal microscopy of meningioma cells (red) in 3D coculture with human cerebral organoids (green) for 30 h demonstrates that M10G cells form tumor spheres adjacent to organoids and M13C cells form tumor spheres that invade organoids. Scale bar, 200 μm. **d** Integrated single cell RNA sequencing analysis of WHO Grade III meningioma cell 2D culture, 12-h and 14-day 3D cultures, 14-day cerebral organoid only culture, and 12-h and 14-day 3D cocultures with cerebral organoids plotted in UMAP space and shaded by sample reveals distinct clusters segregating primarily by sample. **e** UMAP of 2D meningioma cells demonstrates five clusters distinguished by enrichment of extracellular matrix organization and integrin interaction genes (C0), cell proliferation and FOXM1 target genes (C1), metabolism (C2), and a rare subpopulation marked by expression of the ADC high genes *CDH2* and *PTPRZ1* (C4). **f** UMAP of 3D meningioma cells at 12 h and **g** at 14 days reveals persistence of extracellular matrix organization and proliferating meningioma cells, but loss of meningioma cells expressing ADC high genes *CDH2* and *PTPRZ1*. **h** UMAP of meningioma cells in co-culture with human cerebral organoids at 12 h and **i** 14 days demonstrates expression of *CDH2* and *PTPRZ1* in both cell populations, which increasing expression of as *PTPRZ1* over time, as illustrated in feature plots at **j** 12 h and **k** 14 days.

Genetically engineered mouse models designed to recapitulate meningioma biology are confounded by a preponderance of non-meningeal tumors^47,48^, and immunodeficient patient-derived meningioma xenografts are suboptimal for studying cancer cell interactions in the tumor microenvironment. In contrast, organoids derived from human cells can model cancer cell behavior in three dimensions to elucidate how different cell populations interact at the level of single cells^49^. Indeed, we and others have shown that glioblastoma cells can invade and grow in human cerebral organoids, and can generate intra-organoid tumors that closely phenocopy primary tumor cell behavior^42,50,51^. Thus, we hypothesized that coculture of meningioma cells with human cerebral organoids would facilitate real-time investigation of cancer cell interactions in a physiologic microenvironment and reveal gene expression programs that are important for meningioma tumorigenesis. To test this hypothesis, we derived cell lines from spatially defined meningioma samples M10G and M13C, and verified that the CNV profiles of these cells were concordant with their samples of origin (Supplementary Fig. 5). We labeled M10G and M13C meningioma cells with red fluorescent protein, and observed that each grew in a polygonal pattern in 2D culture, but formed tumor spheres in 3D culture conditions (Fig. 5a, b).

To study meningioma tumorigenesis and meningioma cell interaction with the tumor microenvironment, we co-cultured M10G and M13C meningioma cells with human cerebral organoids containing predifferentiated human pluripotent stem cell-derived astrocytes labeled with green fluorescent protein. This organoid model is comprised of structurally complex human astrocytes, which comprise the majority of cell types in the adult human brain, and are capable of producing synapses in high-density organoid coculture^52^. Using live imaging, we found that M10G cells, which were derived from a WHO grade I meningioma, formed tumor spheres at the surface of cerebral organoids (Fig. 5c and Supplementary Movie 1). In contrast, M13C cells, which were derived from a brain-invasive WHO grade III meningioma, formed tumor spheres that invaded cerebral organoids (Fig. 5c and Supplementary Movie 2).

Our data demonstrate that 3D culture of meningioma cells with human cerebral organoids can be used to model meningioma tumorigenesis, and provide a framework for identifying gene expression programs underlying meningioma formation. To do so, we performed single-cell RNA sequencing on meningioma cells derived from a WHO grade III tumor (i) in 2D culture, in 3D culture after (ii) 12 h and (iii) 14 days, in 3D coculture with human cerebral organoids after (iv) 12 h and (v) 14 days, and also on (vi) human cerebral organoids. We performed pooled analysis with batch corrected dimensionality reduction comprising a total of 6817 cells across all six conditions (Supplementary Fig. 6a, b). When evaluating how the uniform manifold approximation and projection (UMAP) clusters were distributed across samples, we found that the clusters predominantly segregated based on culture conditions (Fig. 5d). Three clusters where comprised primarily by 2D meningioma cells enriched for metabolic pathways, cell cycle and FOXM1 target genes, and genes involved in extracellular matrix organization and integrin interaction by gene ontology analysis (Supplementary Fig. 6c). We further identified a cluster comprised by the human cerebral organoid sample, and the genes distinguishing this cluster were subsequently used to distinguish meningioma cells and organoid cells in co-culture conditions (Supplementary Fig. 6c and Supplementary Data 3). Finally, we found distinct clusters of early 3D meningioma cells (12 h), late 3D cultured meningioma cells (14 day), and meningioma cells cocultured with human cerebral organoids (Fig. 5d and Supplementary Fig. 6c).

These data indicate that culture condition is critical for establishing transcriptional identity in meningioma cells. Thus, we performed differential expression analysis on our single cell RNA sequencing data to evaluate differences between 2D, 3D, and cocultured meningioma cells (Supplementary Data 4). To identify mechanisms driving meningioma cell proliferation or tumorigenesis in our model system, we cross-referenced differentially expressed genes from single cells with those from spatially distinct samples with low and high ADC values (Supplementary Data 1). Remarkably, we found that meningioma cells in coculture with human cerebral organoids expressed the greatest number of overlapping genes with high ADC regions in vivo, suggesting that the tumor microenvironment plays an important role in defining meningioma cellular outcomes (Supplementary Data 4). To investigate the expression of genes enriched in regions with elevated ADC in our model system, we analyzed each single-cell RNA sequencing sample individually, and performed gene ontology analyses to identify meningioma cell states (Supplementary Data 5). 2D meningioma cells were subdivided into five clusters including proliferating cells expressing the FOXM1 target genes *CCNB1* and *TOP2A*, cells enriched for metabolic pathways, and cells enriched for extracellular matrix organization and integrin interaction pathways (Fig. 5e). A minority of meningioma cells in 2D culture expressed genes enriched in regions with elevated ADC, such as *CDH2* and *PTPRZ1* (Fig. 5e and Supplementary Fig. 6d). Similarly, dimensionality reduction analysis of both early (Fig. 5f) and late (Fig. 5g) 3D meningioma cells identified shared clusters corresponding to meningioma cells involved in proliferation and extracellular matrix organization, respectively. There was also a meningioma cell cluster enriched for *CDH2* and *PTPRZ1* that was present after 12 h in 3D monoculture, but not after 14 days (Supplementary Fig. 6d), suggesting that neither 2D nor 3D monoculture conditions accurately recapitulate the in vivo molecular heterogeneity of meningioma cells. In contrast, analysis of meningioma cells in co-culture with human cerebral organoids after 12 h (Fig. 5h) and 14 days (Fig. 5i) revealed that organoid derived astrocytes also expressed *CDH2* and *PTPRZ1* (Fig. 5j, i), and that in contrast to monoculture conditions, meningioma cells in co-culture samples maintained *CDH2* expression (Supplementary Fig. 6e) and increased *PTPRZ1* expression over time (Supplementary Fig. 6f). Consistently, both astrocytes and meningioma cells in coculture samples increased expression of the PTPRZ1 ligand *PTN* over time (Supplementary Fig. 6g), which activates PTPRZ1 to promote glioblastoma stem cell renewal and tumor growth^44^. In contrast, *PTN* expression in meningioma cells decreased over time in 3D monoculture conditions (Supplementary Fig. 6g), perhaps explaining the loss of *PTPRZ1* expression from meningioma cells in the absence of a tumor microenvironment (Fig. 5h, i). Indeed, the absence of a tumor microenvironment may also contribute to the development of meningioma cell clusters distinguished by metabolic and extracellular matrix organization pathways in monoculture conditions (Fig. 5e–g), which were not identified in coculture samples (Fig. 5h, i). Nevertheless, these data demonstrate that our novel human cerebral organoid model of meningioma tumorigenesis recapitulates gene expression programs that underlie radiographic heterogeneity in spatially distinct primary meningioma samples (Fig. 3), suggesting that this system may be useful for mechanistic interrogation of meningioma cell proliferation and tumorigenesis.

### CDH2 and PTPRZ1 drive meningioma tumorigenesis

**Fig. 6:**
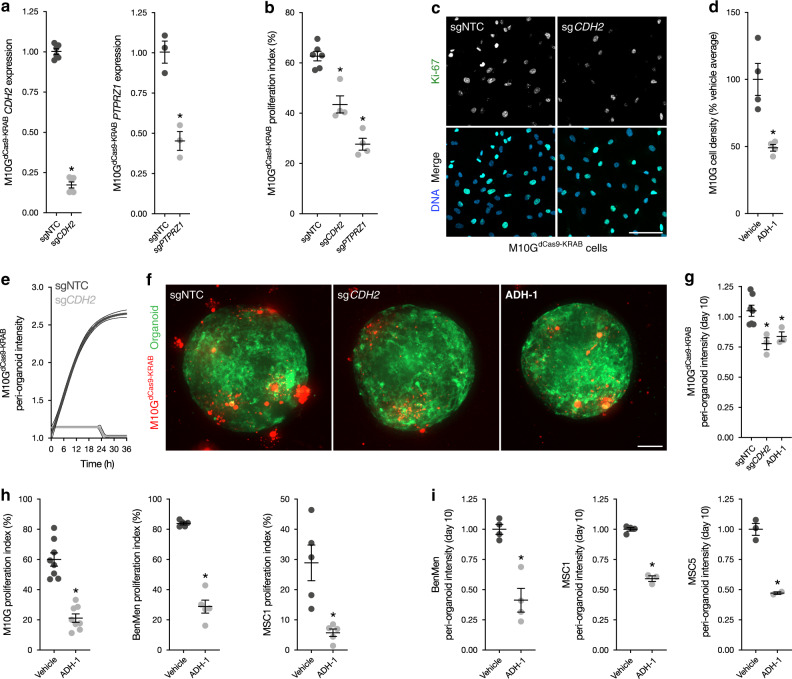
CDH2 and PTPRZ1 drive meningioma cell proliferation and tumorigenesis. **a** qRT-PCR assessment of *CDH2* and *PTPRZ1* expression in M10G^dCas9-KRAB^ meningioma cells after transduction of sgRNAs targeting these genes demonstrates repression of each gene relative to cells transduced with non-targeted control sgRNAs (sgNTC). **b**, **c** Immunofluorescence of Ki-67 (green) in M10G^dCas9-KRAB^ meningioma cells after transduction sgRNAs shows that repression of *CDH2* and *PTPRZ1* blocks meningioma cell proliferation. DNA is marked with Hoeschst (blue). Scale bar, 100 μm. **d** Treatment of M10G cells with the *CDH2* antagonist ADH-1 0.2 mg/ml for 48 h blocks meningioma cell proliferation. **e** Live confocal microscopy of M10G^dCas9-KRAB^ meningioma cells in coculture with human cerebral organoids demonstrates that transduction of sg*CDH2* blocks meningioma tumorigenesis compared to transduction of sgNTC. **f**, **g** Quantification of M10G^dCas9-KRAB^ (red) intensity in coculture with human cerebral organoids (green) after 10 days shows that transduction of sg*CDH2* and treatment with ADH-1 0.2 mg/ml blocks meningioma tumorigenesis. Scale bar, 100 μm. **h** Immunofluorescence of Ki67 in M10G, BenMen, and MSC1 meningioma cells shows that pharmacologic inhibition of *CDH2* with ADH-1 0.2 mg/ml blocks meningioma cell proliferation. **i** Quantification of BenMen, MSC1, and MSC5 intensity in coculture with human cerebral organoids after 10 days shows that treatment with ADH-1 0.2 mg/ml blocks meningioma tumorigenesis. *P ≤ 0.05, two-tailed Student’s unpaired *t* test. Mean ± standard error of the mean (as shown by error bars in **a**, **b**, **d**, **g**, **h**, and **i**) are denoted.

Due to the intrinsic morbidity of salvage surgery and radiotherapy for recurrent meningioma^53–55^, there is an urgent, unmet need for new therapies to treat patients with high grade or recurrent tumors^4,5^. We have shown that meningiomas with elevated ADC are at risk for high grade and recurrence^45,46^, and have identified molecular signatures associated with high ADC values in vivo (Fig. 4) and in vitro (Fig. 5). To determine if genes underlying high ADC values were important for meningioma cell proliferation, we generated meningioma cells derived from the spatially-distinct sample M10G that stably expressed the CRISPRi components dCas9-KRAB^56^, and repressed *CDH2* and *PTPRZ1* by transducing cells with lentiviruses harboring sgRNAs against those genes (Fig. 6a). We performed immunofluorescence for Ki-67 and found that repression of *CDH2* and *PTPRZ1* each attenuated meningioma cell proliferation compared to cells transfected with non-targeting sgRNAs (sgNTC) (Fig. 6b, c).

*CDH2* encodes N-cadherin, a transmembrane glycoprotein that mediates cell-cell adhesion and noncanonical Wnt signaling at adherens junctions^39^. Dysregulated Wnt signaling is implicated in meningioma^19,57,58^, and the *CDH2* small molecule antagonist ADH-1 is both safe and effective at blocking N-cadherin-dependent cancer growth^59–61^. Consistently, we found that ADH-1 blocked the proliferation of M10G meningioma cells in 2D monoculture (Fig. 6d). To determine if inhibition of *CDH2* blocked meningioma tumorigenesis, we cocultured M10G^dCas9-KRAB^ cells, transduced with either sgNTC or sg*CDH2*, with human cerebral organoids, and monitored meningioma cell interactions in the tumor microenvironment using live imaging. We found that repression of *CDH2* blocked meningioma tumorigenesis (Fig. 6e), and moreover, that both genetic and pharmacologic inhibition of *CDH2* with ADH-1 attenuated meningioma cell proliferation in coculture with human cerebral organoids (Fig. 6f, g).

To test the generalizability of pharmacologic inhibition of CDH-2 to block the growth of meningioma cells, we treated primary meningioma cells derived from tumors not included in our cohort of spatially distinct meningioma samples with ADH-1, followed by immunofluorescence for Ki-67 from 2D monocultures (Fig. 6h), and quantification of meningioma tumorigenesis from 3D cocultures with human cerebral organoids (Fig. 6i). These cell lines included BenMen WHO grade I meningioma cells, a previously reported primary meningioma cell line^62^, and MSC1 WHO grade II and MSC5 WHO grade I meningioma cells which we derived de novo and verified that the CNV profiles of these cells were concordant with their samples of origin (Supplementary Fig. 7). Consistent with results using M10G cells, we found that ADH-1 blocked the proliferation (Fig. 6h) and tumorigenesis (Fig. 6i) of all primary meningioma cells tested in monocultures and in cocultures with human cerebral organoids, respectively. In summary, by integrating our bioinformatic, histopathologic and radiologic findings with our human cerebral organoid model of meningioma, we identify *CDH2* and *PTPRZ1* as genes that are enriched in high ADC regions that drive meningioma cell proliferation and tumorigenesis, and represent novel targets for molecular therapy to treat meningioma patients.

## Discussion

We have identified genomic and radiographic determinants of intratumor heterogeneity that elucidate molecular mechanisms underlying meningioma cell proliferation and tumorigenesis. We find that high grade meningiomas are defined by transcriptomic and epigenomic heterogeneity, clonal CNVs, and regions with elevated ADC that distinguish developmental gene expression programs. Among these genes, we find that *FOXM1*, *CDH2*, and *PTPRZ1* are heterogeneously expressed in individual meningiomas. The importance of FOXM1 for high grade meningioma growth is established^19^, but our discovery of spatially distinct patterns of *FOXM1*, *CDH2*, and *PTPRZ1* expression provides a basis for understanding why meningiomas grow asymmetrically. Further, our data suggest that regional meningioma gene expression programs may be driven by compartmentalized genomic rearrangements.

These data can be interpreted according to either the clonal model of cancer formation, or the cancer stem cell hypothesis^63^. The clonal model posits that early during tumor development, a single cell transforms to acquire the potential for unlimited growth, and that its decedents may accumulate additional advantageous mutations over time. We find that meningiomas are characterized by clonal CNVs early in tumor evolution, suggesting that chromosomal structural alterations, particularly loss of chromosome 22q, are an early event during meningioma growth. Importantly, these data validate longstanding assumptions about meningioma tumorigenesis from hereditary cases. Moreover, while it is known that high grade meningiomas have increased genomic instability^12,19^, we show that this process is spatially heterogeneous in individual tumors. Thus, loss of chromosome 22q may represent only one of many genomic drivers that, when combined together in various permutations, may give rise to the multitude of histologic subgroups and clinical presentations of meningioma^3^.

In contrast, the cancer stem cell hypothesis proposes that the cells driving tumor growth are rare and have multipotent potential that manifests through asymmetric divisions. The concept of cancer initiation and progression from primitive embryonal stem cell rests is well established, particularly for pediatric malignancies where histologic, proliferation, differentiation, and gene expression similarities exist between fetal tissues and cancer cells. Our discovery of Wnt and neural crest genes that drive meningioma tumorigenesis and cell proliferation supports the hypothesis that conserved molecular processes that are active during development also underlie meningeal transformation. Consistently, we identify overlapping developmental gene expression programs in meningioma using MR imaging-stratified bulk RNA sequencing, and single cell RNA sequencing of meningioma cells in coculture with human cerebral organoids. The partial concordance of gene expression programs across experimental conditions that we observe, as well as the diverse roles these genes play in both development and cancer, suggest that the identity and timing of gene expression in meningioma may be highly context and culture condition dependent. Indeed, we also identified a pronounced immediate early gene response across meningioma 3D culture and coculture conditions, suggesting contextual reactivation of genes occurs only in a specific set of circumstances in response to critical cellular signaling events. Alternatively, given the range of contexts in which the immediate early gene response is implicated, it is possible that meningiomas may more broadly arise from partial misactivation of developmental gene expression programs driven by upstream signaling events such as receptor tyrosine kinases, bone morphogenetic proteins or Wnt agonizts, similar to Hedgehog pathway misactivation in basal cell carcinoma, medulloblastoma, and other inherited and sporadic malignancies^64^. Further investigation of meningioma tumorigenesis in the context of other cerebral organoid models may shed additional light on these controversies, but in the interim, the data presented here suggest that organoids represent a technologic innovation for understanding the biology of meningioma, a tumor with few tractable model systems.

Numerous prognostic models have been developed to identify meningiomas at risk for recurrence, including those based on histologic features^3^, clinical and demographic characteristics^65^, imaging variables^45^, transcriptomes^19,66,67^, and DNA methylation^15,22,23^. Among these, DNA methylation profiling has emerged as a robust diagnostic and prognostic marker to classify meningiomas and estimate the risk of recurrence. However, clinical DNA methylation profiles are meningioma have been established from a single sample per tumor, raising the possibility that sampling bias could influence the accuracy of methylation-based classification or prognostication. Although hierarchical clustering of DNA methylation profiles grouped spatially distinct samples together according to their tumor of origin in our study (Supplementary Fig. 2), random forest classification of methylation profiles demonstrated a wide range of diagnostic certainty for spatially distinct samples from individual tumors (Supplementary Data 6), consistent with the published finding that regional differences in cell type heterogeneity can influence the accuracy of DNA methylation-based classification of central nervous system tumors^68^. In contrast, it is reassuring that regional histopathologic differences were concordant across spatially distinct samples from each meningioma with respect to WHO grading criteria^3^. Although it possible that this finding might change in a larger dataset, the data presented here validate known patterns of regional differences in histopathologic characteristics and genomic architecture in meningiomas^30–32^. The limitations of our small sample size and referral pattern to our quaternary medical institution also introduces the possibility that the biology we report may be reflective of outliers within the greater population of meningioma patients. Although tumors such as M13 in our study are common at our institution, highly aggressive meningiomas such as this are certainly not representative of average meningioma patients.

In summary, our findings support the large body of literature lending credence to existing histopathologic criteria for meningioma prognostication, and indicate that existing, robust prognostic models should not be discounted when incorporating new technologies into clinical decision making. Rather, our results suggest that new and old models may be integrated to optimize clinical stratification of patients, as we have previously done using clinical, demographic, and imaging characteristics of meningioma patients alongside tumor grade^45,65^. In particular, we suggest that neurosurgeons consider image-guided sampling of regions with elevated ADC for histopathologic evaluation to determine whether they contain features that may predict aggressive clinical behavior that would otherwise go undetected. It is also possible that restricting DNA methylation-based classification of meningiomas to ADC high regions may reduce diagnostic and prognostic heterogeneity. In the interim, our data demonstrate that the molecular programs underlying regions with elevated ADC in high grade meningiomas are potential novel targets for meningioma therapy, and that human cerebral organoids are useful models for investigating meningioma biology and establishing biologic rationale for clinical trials in meningioma patients.

## Methods

### Patients and samples

Patients presenting for resection of meningioma who consented to tumor sampling for research were included in the study, which was approved by the UCSF Institutional Review Board (10-01318 and 17-23196). Cases were selected for inclusion if the meningioma (i) was large enough to obtain multiple samples, (ii) was located in a region facilitating safe intratumor sampling, and (iii) had not undergone preoperative embolization.

Subjects were brought to the operating room, sedated with general anesthesia, and positioned for meningioma resection in a fixed head holder. Preoperative stereotactic MR images were registered to 3D physical space using the stereotactic neuro-navigation system (BrainLab AG^®^; Munich, Germany). Anatomic landmarks were used to confirm the accuracy of registration. Craniotomies were performed in the usual fashion, and once the dura was opened the accuracy of neuro-navigation was confirmed before starting each meningioma resection. Stereotactic samples for multiplatform molecular profiling were selected by the surgeon with the goal of distributing samples evenly across the whole tumor. However, samples were only collected if the surgeon deemed it safe to pause the resection, accurately record the stereotactic coordinates of each sample, and collect tissue for research. To do so, a micropituitary rongeur was used to take 100–200 mg samples from each location. The neuro-navigation probe was subsequently placed in the field at the location where the sample was collected, sample digital imaging and communications in medicine (DICOM) file coordinates were recorded, and a screenshot was captured to corroborate probe location. Meningioma samples were immediately passed off the operating field and placed in liquid nitrogen or cell culture media.

Preoperative stereotactic MR images were imported into 3D Slicer v4.8^69^. The brain and meningioma were individually segmented using the editor module, and the tumor volume was quantified using the label statistics module. DICOM coordinates collected from the stereotactic neuro-navigation system were entered into the markups module and used to generate a 3D model of each meningioma with sample sites.

### Nucleic acid extraction

RNA and DNA were isolated from tumor samples using the All-Prep Universal Kit (QIAGEN, Valencia, CA). In brief, flash frozen tumor samples were thawed in RLT Plus Buffer with beta-mercaptoethanol and were mechanically lysed using a TissueLyzer (QIAGEN) with stainless steel beads at 30 Hz for 90 s. QiaCubes were used for standardized automated nucleic acid extraction per the manufacturer’s protocol (QIAGEN). RNA quality was assessed by chip-based electrophoresis (Agilent Technologies, Waldbronn, Germany), and clean-up was performed as needed using the RNeasy kit (QIAGEN). DNA quality was assessed by spectrophotometry and clean-up was performed as needed using DNA precipitation.

### Bulk RNA sequencing and analysis

Library preparation was performed for 75 meningioma samples with RNA of sufficient quality using the TruSeq RNA Library Prep Kit v2 (RS-122- 2001, Illumina, San Diego, CA) and 50 bp single end reads were sequenced on an Illumina HiSeq 2500. FASTQ file quality control was performed with FASTQC, and reads were filtered to remove bases without an average quality score of 20 within a sliding window of four bases, after trimming of adapter sequences (http://www.bioinformatics.babraham.ac.uk/projects/fastqc/). Mapping to the human reference genome hg19 was performed using HISAT2 with default parameters^70^. Transcripts were calculated in transcripts per million (TPM), and differential expression analysis at a significance level of *q* < 0.1 was performed using DESeq2^71^. The clusterCons package in R was used for hierarchical clustering using the top 2000 most variable genes to determine the optimal number of groups across all samples^72^. Principal component analysis was performed in the statistical environment R version 3.4.3 using the base command ‘prcomp’ with the parameters ‘center = TRUE, scale. = FALSE’ on log2 transformed TPM values with a pseudocount of 1. Genes were ranked by the absolute value of the maximum gene loading scores in principal component analysis space among the first three principal components, which was determined based on diminishing variance explained in principal components four and greater, and gene ontology analysis was performed with the top 250 genes in each PC axis or the full set of differentially expressed genes from DESeq2 when comparing clusters or clinical groups^35–37^.

### DNA methylation arrays and analysis

Methylation analysis was performed according on the Illumina Methylation EPIC Beadchip according to the manufacturer’s instructions. Only probes with detection *P* < 0.05 in all samples were included for further analysis. The minfi Bioconductor^73^ package in R was used for preprocessing, and normalized using functional normalization^74^. Probes were filtered based on the following criteria: (i) removal of probes targeting the X and Y chromosomes (*n* = 11,551), (ii) removal of probes containing a common single nucleotide polymorphism (*n* = 24,536), and (iii) removal of probes not uniquely mapping to the hg19 human reference genome (*n* = 9993). A total of 815,630 probes were retained for further analysis. Principal component analysis was performed using R version 3.4.3 using the base command ‘prcomp’ with the parameters ‘center = TRUE, scale = FALSE’ using *β* values (*β* = methylated/[methylated + unmethylated]). For probe-level differential methylation analysis, the limma Bioconductor package was used to fit a linear model accounting for the paired nature of the data with a FDR < 0.001 considered significant^75^. *β* values (methylated/[methylated + unmethylated]) were used for analyses of methylation levels and *M* values were used for statistical analysis (*M* = log2[methylated/unmethylated])^76^. CNV profiles were generated using the *conumee* package^68^.

### Phylogenetic analysis

A distance matrix using binary CNV calls across all samples was built using the Manhattan distance metric compared to a normal tissue sample for which all CNVs were absent. The ordinary least-squares minimum evolution approach from the *APE* R package was used to generate phylogenetic trees^77^. The total number of CNVs attributed to each branch was annotated.

### Quantitative MR imaging

Brain MR imaging was performed on a 3.0 tesla scanner (Discovery MR750; GE Healthcare, Waukesha, WI) using a neuronavigation protocol that included 3D volumetric anatomic sequences, axial diffusion tensor imaging (DTI), and axial arterial spin labeling (ASL) perfusion imaging. Preoperative MR images were imported into 3D Slicer v4.8.0, and DICOM coordinates collected from the stereotactic neuronavigation system were entered to generate a 3D model of the tumor with sample sites.

For radiologic characterization of spatially distinct samples, regions of interest were created around each point and used for downstream image analysis. DTI and ASL images were aligned to 3D T1 post-contrast images using FMRIB’s Linear Image Registration Tool (http://fsl.fmrib.ox.ac.uk). Voxel-by-voxel ADC maps were generated from the DTI imaging dataset, and ADC high and ADC low regions were defined by dichotomizing samples at the mean ADC across all samples (threshold 0.8 × 10^−3^). For ASL perfusion calculations, cerebral blood flow (CBF) maps were created on a voxel-by-voxel basis. Totally, 100 mm^3^ spherical regions of interest were delineated at each stereotactic sampling site to allow for collection of quantitative diffusion and perfusion metrics allowing for tissue shifts and sampling margin of error.

### Histology, live microscopy, and immunofluorescence

Formalin-fixed and paraffin-embedded, 4 μm thick, hematoxylin and eosin-stained sections from the permanent histopathologic record of each case were reviewed and compared to frozen 10 μm sections from stereotactic research samples by a neuropathologist who was blinded to sample location, ADC value, CNV, RNA sequencing, and DNA methylation profile.

Immunofluorescence for Ki-67 from meningioma cell cultures was performed on glass coverslips. Cells were fixed in 4% paraformaldehyde, blocked in 2.5% fetal bovine serun (FBS), 200 mM glycine and 0.1% Triton X-100 in phosphate-buffered saline for 30 min at room temperature (Thermo Fisher Scientific, Waltham, MA), and labeled with anti-Ki-67 (Abcam, Cambridge, UK, ab15580; labeling validated by knockout) primary antibodies at 4 °C overnight. Cells were labeled with Alexa Fluor secondary antibodies and Hoescht to mark DNA (Life Technologies, Carlsbad, CA, H3570) for 1 h at room temperature, and were mounted in ProLong Diamond Antifade Mountant (Thermo Fisher Scientific). Immunofluorescence for Ki-67 and FOXM1 from frozen 10 μm meningioma sections was performed with MIB1 (Ventana Medical Systems, Santa Clara, CA, 790–4286; labeling validated using immunizing peptide) and anti-FOXM1 (Abcam, ab207298; labeling validated using human colon and colon cancer tissues) primary antibodies at room temperature for 32 min, followed by Alexa Fluor secondary antibodies and DAPI to mark DNA (Life Technologies) for 20 min at room temperature. Immunofluorescence microscopy was performed on a Zeiss LSM 800 confocal laser scanning microscope with a PlanApo 20× air objective. Images were processed and quantified from no fewer than three distinct regions per coverslip using ImageJ. Observers were blinded to conditions, and Ki-67 or FOXM1 labeling was quantified and normalized to the number of nuclei in each field of view. Primary antibodies were used at a dilution of 1:100, and secondary antibodies were used at a dilution of 1:500.

A Zeiss Cell Observer Spinning Disc Confocal microscope (Carl Zeiss AG, Oberkochen, Germany) fitted with a temperature and carbon dioxide-controlled chamber was used to record live interactions of meningioma cells with cerebral organoids. Organoids were imaged every 5 min for a 36 h period, starting at the time of coculture initiation, using a 10× objective with 0.4 NA.

### Primary meningioma cell and cerebral organoid culture

Primary human meningioma cell lines were derived from fresh meningioma resection specimens by mechanically mincing approximately 100 mg of tumor tissue in HBSS and then plating in media with a 1:1 ratio of DMEM/F12 (Life Technologies, #10565) and Neurobasal medium (Life Technologies, #21103), supplemented with 5% FBS (Life Technologies, #16141), B-27 supplement without vitamin A (Life Technologies, #12587), N-2 supplement (Life Technologies, #17502), 1X GlutaMAX (Life Technologies, #35050), 1 mM NEAA (Life Technologies, #11140), 100 U/mL Anti-Anti (Life Technologies, #15240), 20 ng/mL EGF (R&D systems, Minneapolis, MN, #236-EG), 20 ng/mL FGF2 (Peprotech, Rocky Hill, NJ, #100-18 C). Cell lines were grown to confluence, analyzed by DNA methylation profiling to confirm CNV concordance with resection specimens, and transduced with tdTomato (M10G) or mScarlet (M13C, BenMen, MSC1, and MSC5) red fluorescent protein. The primary human meningioma BenMen cell line was originally derived from a meningothelial meningioma and transfected with hTERT to achieve immortalization^62^. BenMen cells were cultured in DMEM (Life Technologies, #10313021) supplemented with 10% FBS and 1× GlutaMAX.

Human cerebral organoids were created from astrocytes induced from pluripotent human stem cells^52,78,79^. In brief, free floating neuroepithelial aggregates from WTC11 cells were induced by dual SAMD inhibition with SB431542 and DMH-1 (2 µM each, Tocris, #HY-10431 and #4126, respectively). On day 5, aggregates were seeded to 100 ng/mL Matrigel (Corning, Corning, NY, #356231) coated plates and cultured in DMEM/F12, supplemented with 2 µg/mL Heparin (Sigma, St. Louis, MO, #H3149), B-27 supplement without vitamin A, N-2 supplement, and 100 U mL^−1^ Anti-Anti, referred to as NPC medium. Between 5 and 7 days after attachment, rosettes were mechanically isolated and transferred to a culture flask and maintained in NPC medium for 7 days. Therefore, rosette aggregates were further cultured in 10 ng/ml EGF and 10 ng/ml FGF2 to induce astroglial differentiation over the course of approximately 12 months. Prior to organoid formation, cells were transduced with mGFP (Addgene, Watertown, MA, #22479).

For coculture experiments, premature astroglial cells were dissociated with StemPro Accutase (Thermo Fisher Scientific, #A1110501) and 7000 cells were seeded into each well of a PrimeSurface ultra-low attachment V-shaped 96 well plate (S-Bio, Hudson, NH, #MS-9096VZ). On day 5, mono-organoids were transferred to a spheroid microplate (Corning, #4515) prior to adding meningioma cells. Meningioma cells were dissociated with StemPro Accutase, and 2000 meningioma cells were added to each well of mono-organoids and maintained for 14 days in NPC medium supplemented with 10 ng/mL EGF and 10 ng/mL FGF2.

### Single-cell RNA sequencing

Single-cell suspensions of were generated using Accutase (Thermo Fisher Scientific) and processed for single cell RNA-seq using the Chromium Single Cell 3′ Library & Gel Bead Kit v3 on a 10× Chromium controller (10× Genomics) using the manufacturer recommended default protocol and settings, at a target cell recovery of 4000 cells per sample. 100 base pair paired end reads were sequenced on an Illumina NovaSeq at the Center for Advanced Technology at the University of California San Francisco, and the resulting FASTQ files were processed using the CellRanger analysis suite (https://github.com/10XGenomics/cellranger) for alignment to the hg38 reference genome, identification of empty droplets, and determination of the count threshold for further analysis. A cell quality filter of greater than 500 features but fewer than 10,000 features per cell, and less than 10% of read counts attributed to mitochondrial genes, was used. Single cell UMI count data were preprocessed using log normalization, identification of top 2000 variable genes, and gene by gene scaling based on regression of UMI count and percentage of reads attributed to mitochondrial genes per cell were performed in Suerat 3.0^80^. Dimensionality reduction was performed using principal component analysis, and a K-nearest neighbor graph was generated using the first ten principal components. Louvain clustering was performed using a resolution of 0.3. Uniform Manifold Approximation and Projection (UMAP) was performed on the reduced data with a minimum distance metric of 0.2. Marker selection was performed using default settings in Seurat 3.0^80^.

### CRISPR interference and pharmacology

Lentiviral particles containing pMH0001 (UCOE-SFFV-dCas9-BFP-KRAB, Addgene #85969)^81^ were produced by transfecting HEK293T cells with standard packaging vectors using the *Trans*IT-Lenti Transfection Reagent (Mirus Bio, Madison, WI, MIR 6605). M10G cells were stably transduced with these lentiviral particles to generate M10G^dCas9-KRAB^ cells. Successfully transduced cells were isolated through selection for BFP expression cells using fluorescence activated cell sorting on a Sony SH800.

Single-guide RNA (sgRNA) protospacer sequences were individually cloned into the pCRISPRia-v2 vector (Addgene plasmid #84832), between the BstXI and BlpI sites, by ligation^82^. Each vector was verified by Sanger sequencing of the protospacer. Two sgRNA expression vectors with different protospacers were cloned for non-targeting control sgRNAs (GCTGCATGGGGCGCGAATCA and GTGCACCCGGCTAGGACCGG) and *CDH2* (GGGGCCGAGCGAAGAGCCGG and GGAGGGGCCGAGCGAAGAGC), and results were pooled for analysis. One protospacer was cloned to target *PTPRZ1* (GAGCCGAGGCGCATGTCCTC). Lentivirus was generated as described above for each sgRNA expression vector, and M10G^dCas9-KRAB^ cells were independently transduced with lentivirus from each sgRNA expression vector, then selected to purity using 20 µg/mL puromycin over 7 days.

For meningioma cell proliferation assays, in 2D, M10G^dCas9-KRAB^ cells were counted and 25,000 cells were plated per well in 24-well plates with glass coverslips. Cells were treated with 0.2 mg/mL of ADH-1 trifluoroacetate (Monmouth Junction, NJ, HY-13541A, validated by mass spectrometry) or an equivalent volume of DMSO 24 h after plating. Cells were assessed and immunofluorescence was performed 72 h after ADH-1 treatment. For 3D meningioma cell and organoid coculture experiments, media containing ADH-1 or DMSO at the same concentrations used for 2D assays were replaced every 3 days.

### Quantitative reverse transcriptase polymerase chain reaction

RNA was isolated from meningioma cell lines as described above, and cDNA was synthesized using the iScript cDNA Synthesis Kit (Bio-Rad, Hercules, CA) per the manufacturer’s protocol. Target genes were amplified using PowerUp SYBR Green Master Mix and a QuantStudio 6 thermocycler (Thermo Fisher Scientific). Gene expression was calculated using the ΔΔCt method, with normalization to *GAPDH* (sense: 5′-CTTCACCACCATGGAGAAGGC-3′, antisense: 5′-GGCATGGACTGTGGTCATGAG-3′). Target gene primers included *CDH2* (sense: 5′-TCAGGCGTCTGTAGAGGCTT-3′, antisense: 5′-ATGCACATCCTTCGATAAGACTG-3′) and *PTPRZ1* 5′-GCTTTGATGCGGACCGATTTT-3′, antisense: 5′-ACGACTAACACTTTCGACTCCA-3′).

### Statistics and reproducibility

All experiments were performed at least three times with similar results, including no fewer than three organoid/meningioma cell cocultures for each condition. Histograms and scatter plots show mean ± standard error of the mean, or mean ± 95% confidence interval, as indicated. Two-tailed Student’s unpaired *t* tests, and one-way ANOVA, were used to compare groups as indicated, with statistical significance, as denoted by *in figures, defined as *P* ≤ 0.05.

### Reporting summary

Further information on research design is available in the Nature Research Reporting Summary linked to this article.

## Supplementary information

Supplementary Information

Description of Additional Supplementary Files

Supplementary Data 1

Supplementary Data 2

Supplementary Data 3

Supplementary Data 4

Supplementary Data 5

Supplementary Data 6

Supplementary Movie 1

Supplementary Movie 2

Reporting Summary

## Data Availability

DNA methylation and RNA sequencing data that support the findings of this study have been deposited in the NCBI Gene Expression Omnibus (https://www.ncbi.nlm.nih.gov/geo/) under the following accession numbers: GSE151067 (DNA methylation) and GSE151921 (RNA sequencing). Single-cell RNA-seq raw data is available at the Sequence Read Archive (https://www.ncbi.nlm.nih.gov/sra) under project accession PRJNA660307. The remaining data are available within the Article, Supplementary Information, or available from the author upon request.
