# Supplementary material for: Multiplatform genomic profiling and magnetic resonance imaging identify mechanisms underlying intratumor heterogeneity in meningioma
Source: Nat Commun. 2020 Sep 23;11:4803. doi: 10.1038/s41467-020-18582-7 (PMC7511976; doi:10.1038/s41467-020-18582-7)
Supplement: Supplementary file 1 — Supplementary Information [file 41467_2020_18582_MOESM1_ESM.pdf]

Multiplatform genomic profiling and magnetic resonance imaging identify mechanisms underlying intratumor heterogeneity in meningioma

Magill and Vasudevan et al.

Supplementary Figure 1. RNA sequencing reveals transcriptomic intratumor heterogeneity in meningioma.

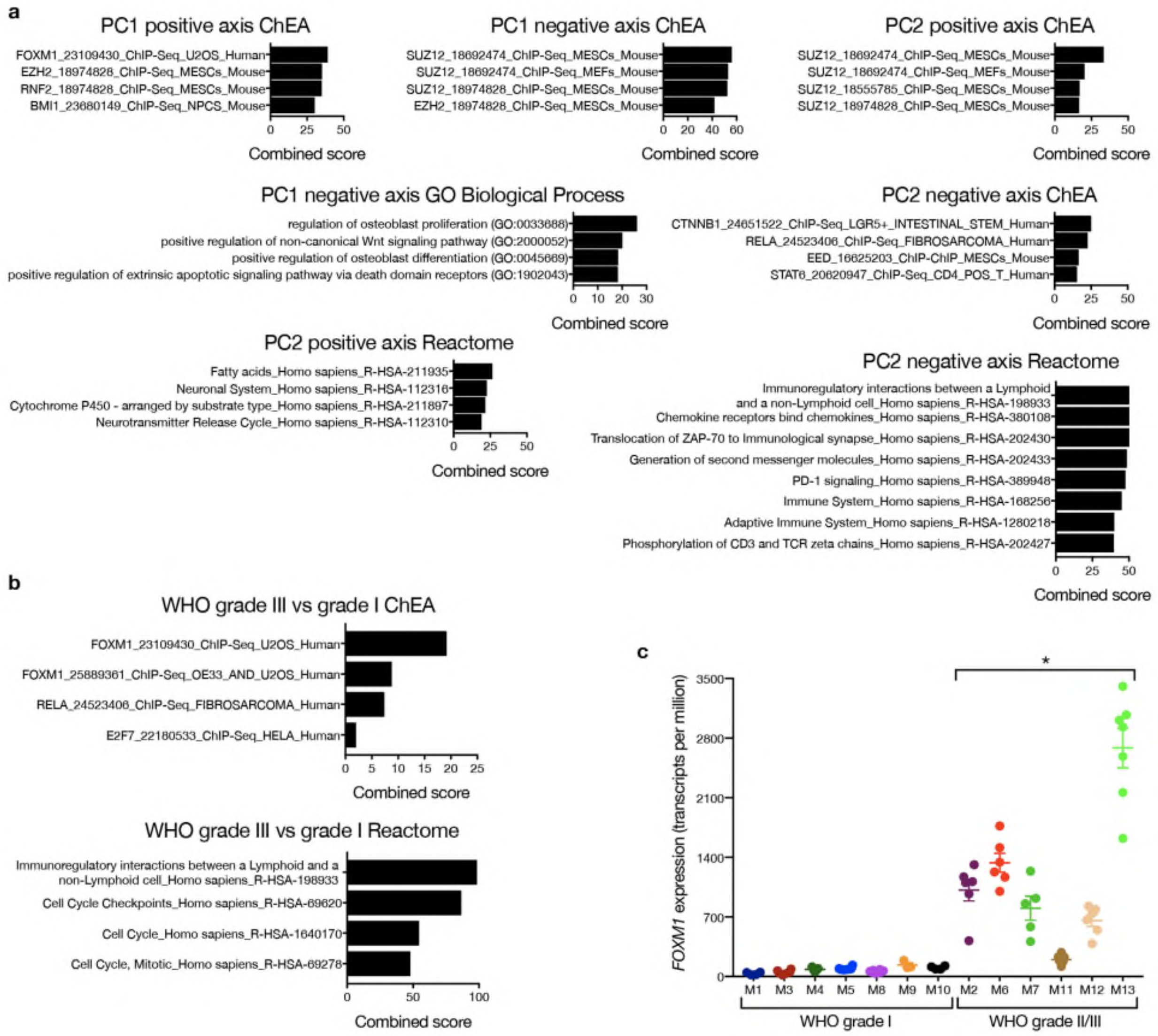

Supplementary Figure 1. RNA sequencing reveals transcriptomic intratumor molecular heterogeneity in meningioma. (a) Gene ontology analysis demonstrates distinct enrichment patterns in spatially distinct meningioma samples that segregate WHO grade I from grade II and grade III tumors. (b) Gene ontology analysis shows that WHO grade III meningiomas are enriched in FOXM1 targets, cell cycle effectors and immunologic signaling relative to WHO grade I meningiomas. (c) RNA sequencing demonstrates that WHO grade II and grade III meningiomas are enriched in *FOXM1* transcripts relative to WHO grade I meningiomas. *P≤0.05, two-tailed Student's unpaired t test. Mean ± standard error of the mean (as shown by

error bars) are denoted. Samples from spatially-distinct meningioma samples are color-coordinated by tumor-of-origin.

Supplementary Figure 2. DNA methylation profiling reveals epigenomic intratumor heterogeneity in meningioma.

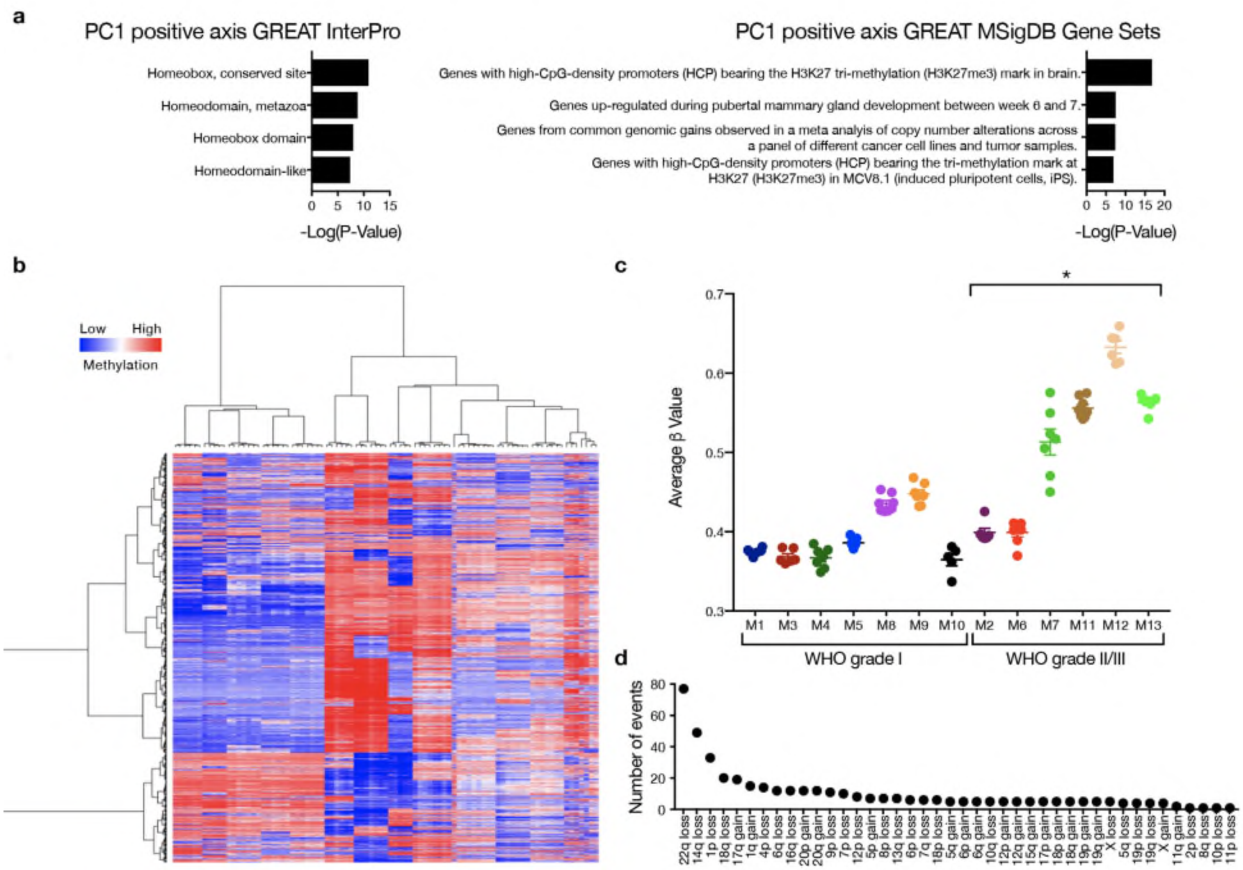

Supplementary Figure 2. DNA methylation profiling reveals epigenomic intratumor molecular heterogeneity in meningioma. (a) Gene ontology analysis demonstrates that spatially distinct meningioma samples from WHO grade II and grade III tumors included in this study are unified by methylation of Homeobox genes and trimethylation of Histone 3 lysine 27. (b) Hierarchical clustering using the top 2000 most variable genes from DNA methylation profiling identifies 2 clusters of samples. (c) WHO grades II and III meningiomas have higher average β methylation values than WHO grade I meningiomas. Mean ± standard error of the mean (as shown by error bars) are denoted. (d) Copy number variants derived from DNA methylation analysis reveals common and rare chromosomal events in meningiomas samples, the former of which are notable for loss of 22q, 1p and 18q, and gain of 17q and 1q. *P≤0.05, two-tailed Student's unpaired t test. Samples from spatially-distinct meningioma samples are color-coordinated by tumor-of-origin.

Supplementary Figure 3. CNVs and molecular heterogeneity in spatially-distinct meningioma samples.

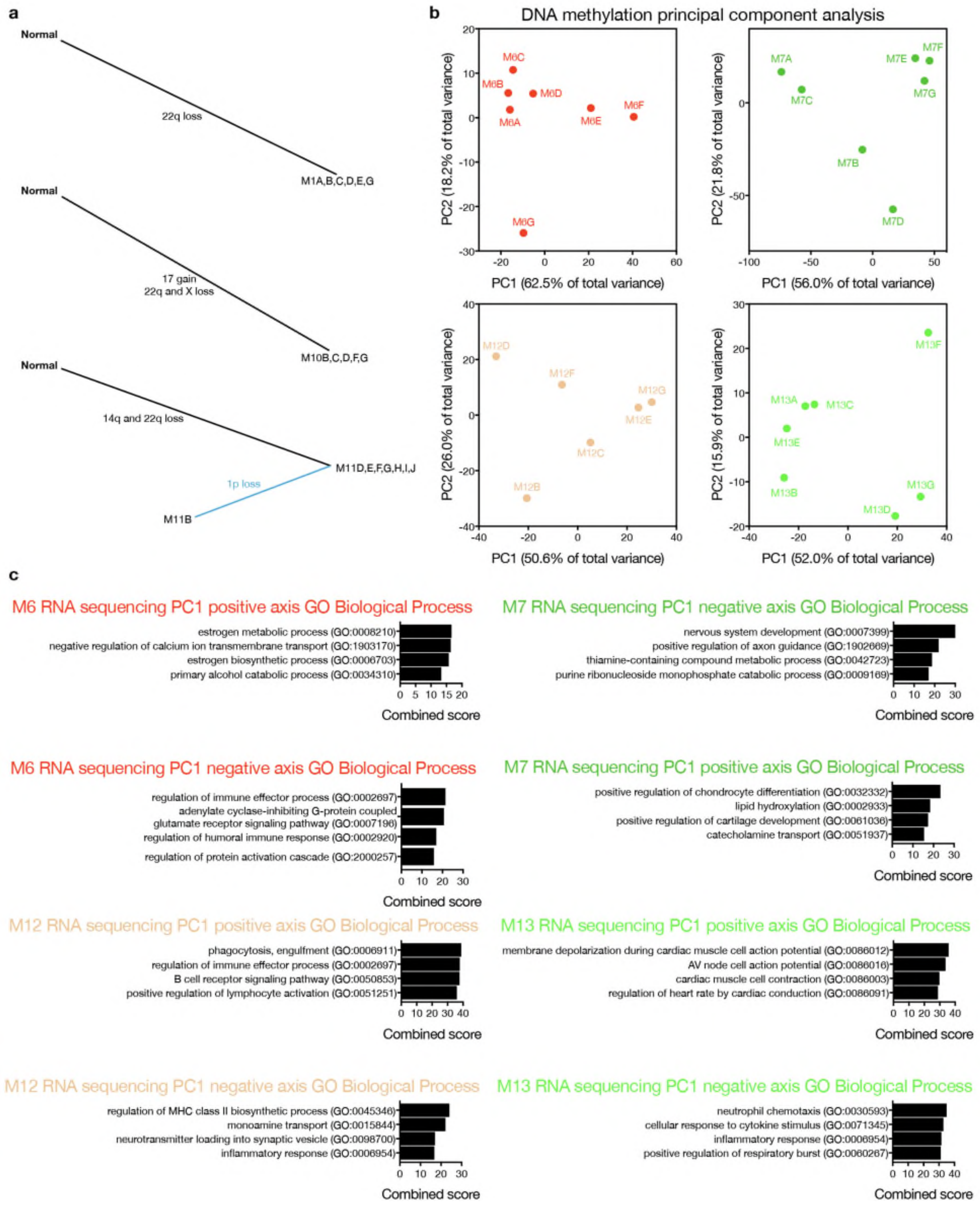

Supplementary Figure 3. CNVs and molecular heterogeneity in spatially-distinct meningioma samples. (a) Intratumor phylogenies based on clonal ordering of copy number variants derived

from DNA methylation profiling of samples from meningiomas M1, M10 and M11 corroborate that chromosomal structural alterations are an early event during meningiomas growth. Clonal variants are shown in black, and shared variants are shown in blue. (b) DNA methylation profiling principal component (PC) analysis reveals that approximately 69-81% of variation among samples from meningiomas M6, M7, M12 and M13 is explained by the first two principal components. (c) Gene ontology analysis from RNA sequencing reveals that differences in immune, GPCR and hormone signaling, nervous system and mesenchymal processes delineate spatially distinct samples from meningiomas M6, M7, M12 and M13. Samples from spatially-distinct meningioma samples are color-coordinated by tumor-of-origin.

Supplementary Figure 4. Intratumor radiographic and histologic heterogeneity in meningioma.

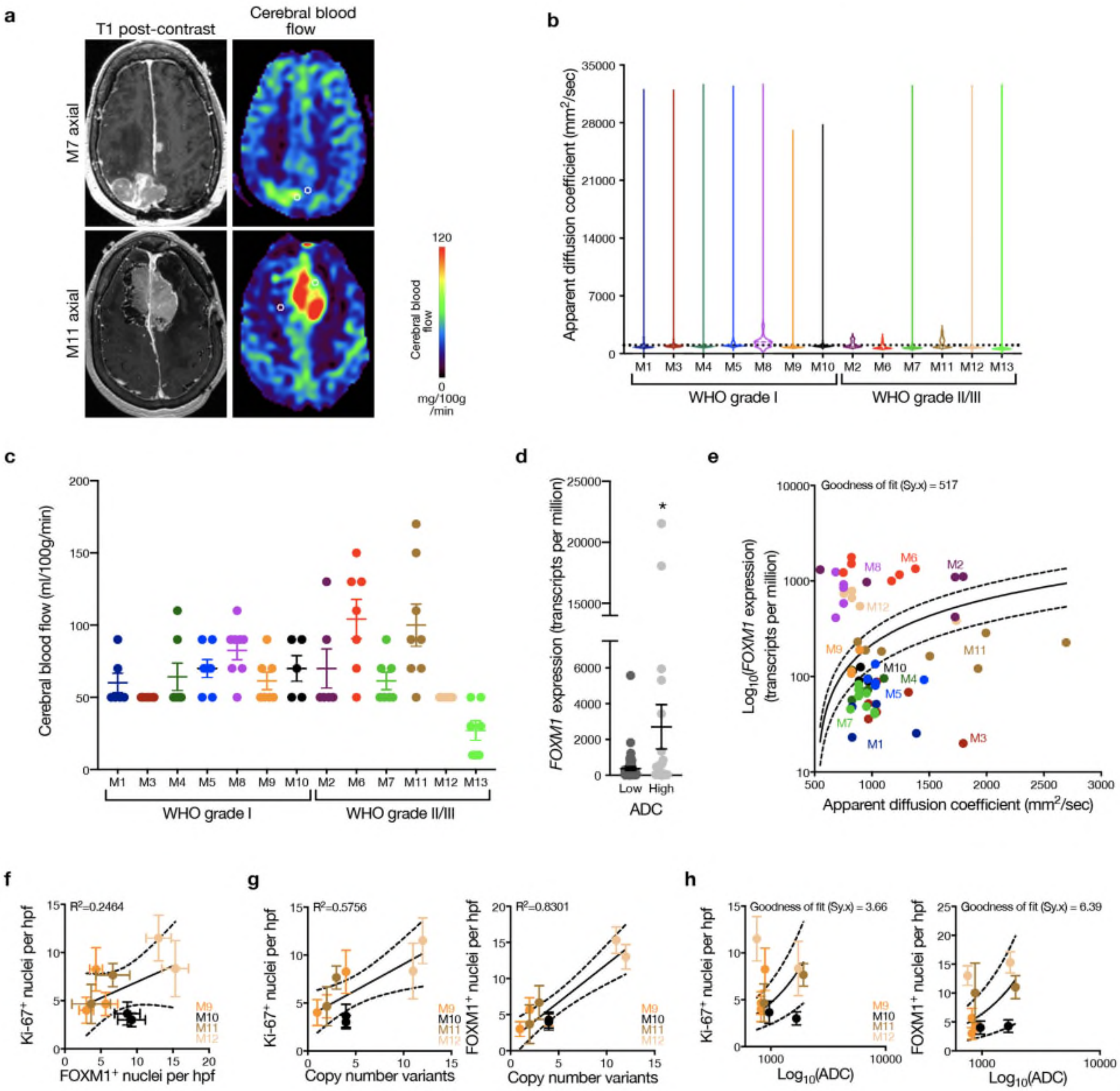

Supplementary Figure 4. Intrameningioma radiographic and histologic heterogeneity in meningioma. (a) Preoperative magnetic resonance imaging of cerebral blood flow. White circles indicate spatially distinct sample locations from meningiomas M7 and M11. (b) Quantitative ADC levels throughout the whole tumor from each case included in this study shows that some high grade meningioma may have low mean ADC values, reflective of high cellularity. (c) Quantitative cerebral blood flow of intratumor samples fails to reveal robust differences in the average or distribution of cerebral blood flow measurements between WHO grade I and WHO grade II and grade III meningiomas. Mean ± standard error of the mean (as shown by error bars)

are denoted. (d) RNA sequencing demonstrates enrichment of *FOXM1* transcripts in ADC high samples relative to ADC low samples of WHO grade II and grade III meningiomas. *P≤0.05, two-tailed Student's unpaired t test. Mean ± standard error of the mean (as shown by error bars) are denoted. (e) RNA sequencing demonstrates expression of *FOXM1* transcripts correlates to ADC levels of spatially-distinct meningioma samples, excluding samples from M13, which demonstrated very high FOXM1 protein expression. (f, g) Quantitative immunofluorescence validates the positive associations between Ki-67 and FOXM1, and each of these with CNVs from spatially-distinct meningioma samples, even in the absence of samples from M13. Mean ± standard error of the mean (as shown by error bars) are denoted. (h) Associations between Ki-67 labeling index, FOXM1 immunofluorescence, and regional ADC were less strong in the absence of spatially-distinct samples from M13. Mean ± standard error of the mean (as shown by error bars) are denoted. Samples from spatially-distinct meningioma samples are color-coordinated by tumor-of-origin.

Supplementary Figure 5. Primary meningioma cell CNV profiles in comparison to spatially distinct samples of origin.

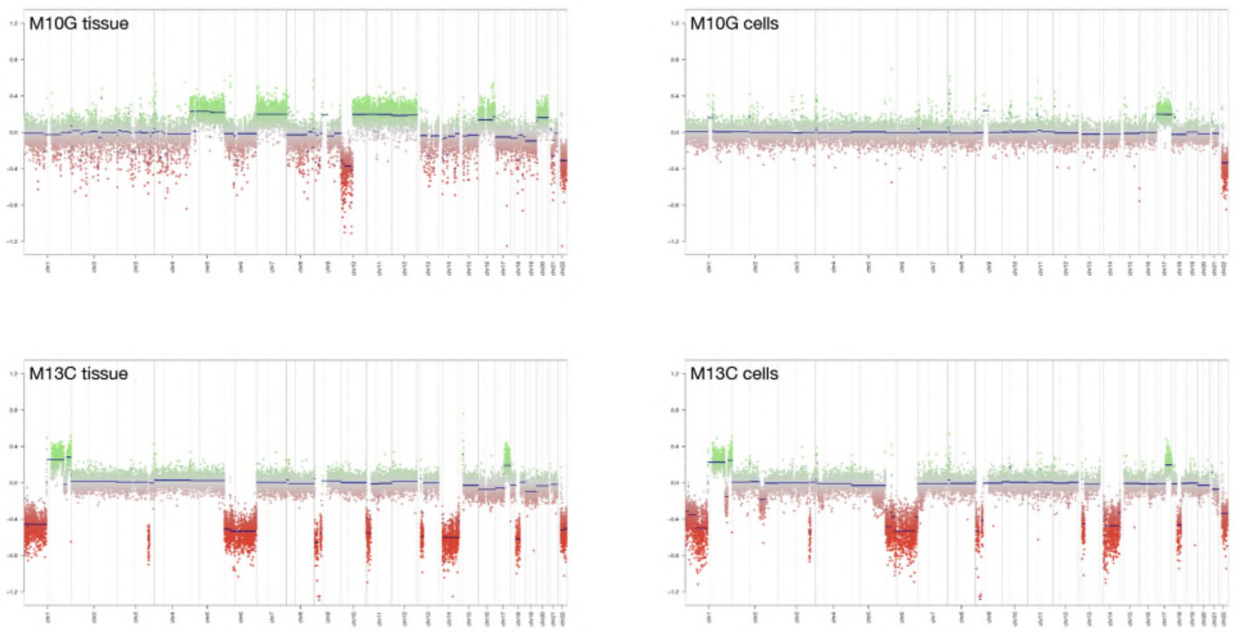

Supplementary Figure 5. Primary meningioma cell CNV profiles in comparison to spatially distinct samples of origin. Copy number variants (CNVs) for each meningioma cell line and sample were determined from DNA methylation profiling. Both M10G and M13C cells retain critical CNVs associated with meningiomas, including loss of chromosome 22q harboring the *NF2* tumor suppressor gene. M13G cells additionally retain the majority of other CNVs found in the tissue sample, while the CNV profile of M10G cells suggests outgrowth of a subclone from within the tissue sample.

Supplementary Figure 6. Meningioma cell and human cerebral organoid single cell RNA sequencing.

a

scRNA-seq clusters	Cells
C0	1704
C1	1625
C2	1065
C3	788
C4	716
C5	593
C6	326
Total	6817

b

	scRNA-seq clusters						
**Sample**	**C0**	**C1**	**C2**	**C3**	**C4**	**C5**	**C6**
Organoids	4	686	3	9	1	18	0
2D meningioma cells	1586	0	819	40	1	0	283
3D meningioma cells 12h	83	1	66	582	13	0	30
3D meningioma cells 14d	25	7	138	122	677	14	1
Meningioma cells and organoids 12h	4	566	29	19	2	161	12
Meningioma cells and organoids 14d	2	365	10	16	22	400	0
Total	1704	1625	1065	788	716	593	326

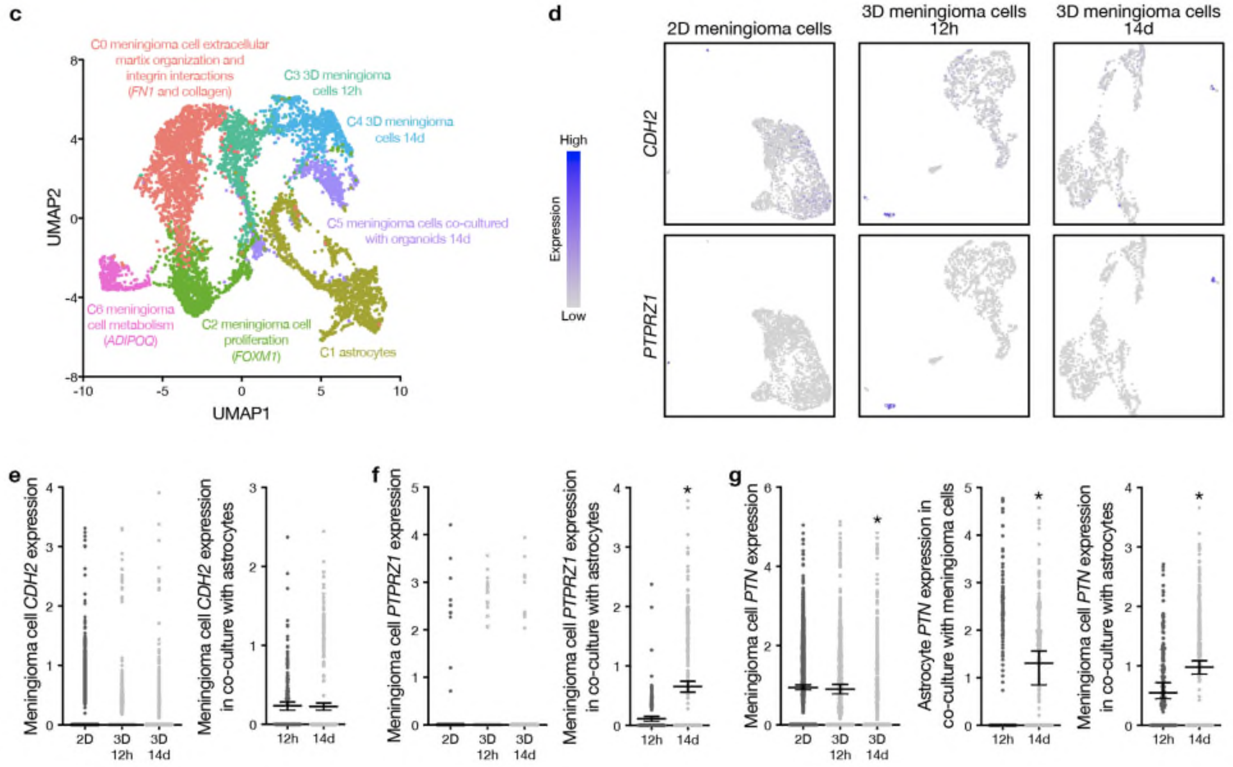

Supplementary Figure 6. Meningioma cell and human cerebral organoid single cell RNA sequencing. (a, b) Cell counts across clusters from single cell RNA sequencing (scRNA-seq) of WHO Grade III meningioma cells and human cerebral organoids across all conditions. (c) Integrated single cell RNA sequencing analysis of WHO Grade III meningioma cell 2D culture, 12 hour and 14-day 3D cultures, 14 day cerebral organoid only culture, and 12 hour and 14 day 3D co-cultures with cerebral organoids plotted in UMAP space and shaded by cluster reveals distinct clusters segregating primarily by sample. (d) Feature plots of 2D and 3D monoculture conditions demonstrate that *CDH2* and *PTPRZ1* are only minimally expressed in these samples. (e) Meningioma cell expression of *CDH2* in 2D, 3D, and co-culture conditions demonstrates that meningioma cells co-cultured with human cerebral organoids express and maintain expression of *CDH2* over time. (f) Meningioma cell expression of *PTPRZ1* in 2D, 3D, and co-culture

conditions demonstrates that meningioma cells co-cultured with human cerebral organoids express and increase expression of *PTPRZ1* over time. (g) Meningioma cell and astrocyte expression of *PTN* in 2D, 3D, and co-culture conditions demonstrates that astrocytes and meningioma cells in co-culture conditions increase expression of *PTN* over time. In contrast, *PTN* decreases over time in meningioma cells in 3D monoculture conditions. *P≤0.05, one-way ANOVA. Mean ± 95% confidence interval (as shown by error bars) are denoted for each plot (e-g). Plots without overtly evident mean and confidence intervals have a mean that approaches zero.

Supplementary Figure 7. Primary meningioma cell CNV profiles in comparison to meningioma samples of origin.

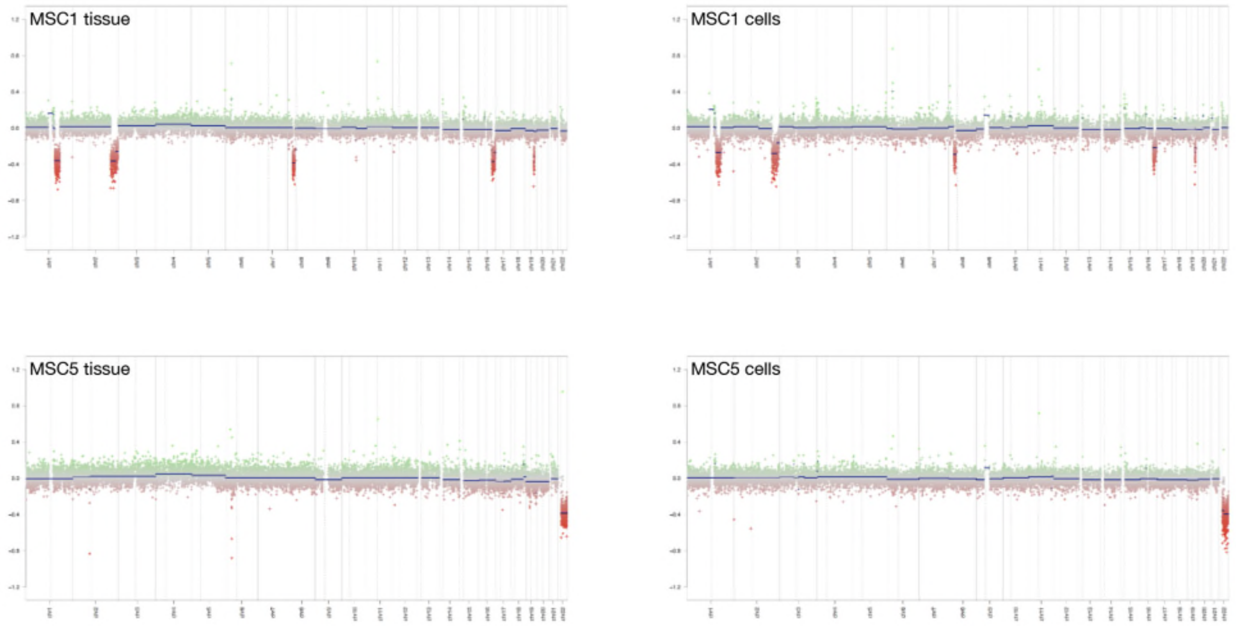

Supplementary Figure 7. Primary meningioma cell CNV profiles in comparison to meningioma samples of origin. Copy number variants (CNVs) for meningioma cell lines and samples from tumors that were not included in our spatially distinct meningioma sample cohort, as determined from DNA methylation profiling. Both MSC1 and MSC5 cells retain critical CNVs associated with meningiomas, including loss of chromosome 22q harboring the *NF2* tumor suppressor gene in MSC5.

Supplementary Table 1. Patient, meningioma and sample characteristics.

**Summary Statistics**	
Patients	13
Median age (range)	65 years (52-79 years)
Male:Female (ratio)	4:9 (1:2.3)
Asian:Caucasian (ratio)	2:11 (1:5.5)
Primary:Recurrent (ratio)	6:7 (1:1.2)
Median size (range)	17 cm^3 (4.7-100 cm^3)
Extent of resection	
Gross total	11 (85%)
Near total	2 (15%)
WHO grade	
I	7 (54%)
II (atypical)	3 (23%)
III (anaplastic)	3 (23%)
Total Samples	86
Median samples per meningioma (range)	7 (5-8)
Median intrameningioma sample separation (range)	14 mm (2.5-177 mm)
Median sample distance from meningioma centroid (range)	13 mm (2.5-172 mm)
Median sample distance from meningioma origin (range)	15 mm (2.3-178 mm)
Median RNA sequencing analyses per meningioma (range)	6 (3-8)
Median DNA methylation analyses per meningioma (range)	6 (5-8)

Patients and meningiomas

Case	*Prior Treatments*	*Location*	*Size (cm^3)*	*EOR*	*Grade*	*Subtype*
M1	Primary	Frontal, parasagittal	15.0	GTR	I	Psammomatous
M2	Rxn x2, SRS	Frontal, parasagittal	4.7	GTR	II	Meningothelial
M3	Primary	Frontal, parasagittal	18.2	GTR	I	Fibrous
M4	Primary	Parietal, parasagittal	16.8	GTR	I	Fibrous

M5	Primary	Frontal, falx	93.3	GTR	I	Psammomatous
M6	Rxn x1	Frontal, parasagittal	13.3	GTR	III	Psammomatous
M7	Rxn x2, SRS, WBRT	Parietal, parasagittal; falco-tentorial	51.6	NTR	III	Meningothelial
M8	Rxn x1	Frontal, parasagittal	99.7	GTR	I	Meningothelial
M9	Primary	Frontal, parasagittal	15.4	GTR	I	Fibrous
M10	Primary	Parietal, falx	17.1	GTR	I	Transitional
M11	Rxn x1	Frontal, parasagittal	74.9	NTR	II	Meningothelial
M12	Rxn x3, EBRT, SRS	Parietal, parasagittal	15.1	GTR	II	Meningothelial
M13	Rxn x4, EBRT, SRS, Brachy	Parietal, parasagittal	21.1	GTR	III	Anaplastic

Samples

Case	*Samples*	*Median separation (range)*	*RNA sequencing*	*DNA methylation*
M1	6	12 mm (6.2-31 mm)	4	7
M2	7	13 mm (2.5-16 mm)	6	6
M3	6	12 mm (6.4-20 mm)	6	6
M4	7	12 mm (4.6-18 mm)	7	7
M5	7	33 mm (8.9-46 mm)	6	6
M6	7	13 mm (4.5-22 mm)	6	7
M7	5	40 mm (8.4-73 mm)	5	7
M8	8	27 mm (7.7-81 mm)	8	8
M9	7	22 mm (2.9 -177 mm)	3	7
M10	5	24 mm (9.6-37 mm)	4	5
M11	8	28 mm (13-47 mm)	7	7
M12	7	14 mm (9-29 mm)	6	6
M13	6	13 mm (3.6-21 mm)	7	7

Extent of resection, EOR; external beam radiotherapy, EBRT; gross total resection, GTR; interstitial brachytherapy, Brachy; near total resection, NTR; resection, Rxn; stereotactic radiosurgery, SRS; year old, yo; whole brain radiotherapy, WBRT.
