# Supplementary material for: Multiplatform genomic profiling and magnetic resonance imaging identify mechanisms underlying intratumor heterogeneity in meningioma
Source: Nat Commun. 2020 Sep 23;11:4803. doi: 10.1038/s41467-020-18582-7 (PMC7511976; doi:10.1038/s41467-020-18582-7)
Supplement: Supplementary file 2 — Description of Additional Supplementary Files [file 41467_2020_18582_MOESM2_ESM.pdf]

Description of Additional Supplementary Files

File name: *Supplementary Data 1*

Description: *RNA sequencing of spatially-defined meningioma samples according to ADC values.* P-values are Bonferroni corrected and two-sided.

File name: *Supplementary Data 2*

Description: *ADC and CNV characteristics of spatially-defined meningioma samples analyzed by histopathology.*

File name: *Supplementary Data 3*

Description: *In vitro meningioma and organoid single cell RNA sequencing cluster marker genes.* P-values are Bonferroni corrected and two-sided.

File name: *Supplementary Data 4*

Description: *Differentially expressed genes among in vitro conditions compared to in vivo gene expression stratified by ADC level.* P-values are Bonferroni corrected and two-sided.

File name: *Supplementary Data 5*

Description: *In vitro meningioma and organoid single cell RNA sequencing cluster gene ontologies.*

File name: *Supplementary Data 6*

CNV totals and random forest classification of spatially-defined meningioma samples.

File name: *Supplementary Movie 1*

Description: *3D co-culture of M10G WHO grade I meningioma cells with human cerebral organoids.*

File name: *Supplementary Movie 2*

Description: *3D co-culture of M13C brain-invasive WHO grade III meningioma cells with human cerebral organoids.*
