# Supplementary material for: Multiplatform genomic profiling and magnetic resonance imaging identify mechanisms underlying intratumor heterogeneity in meningioma
Source: Nat Commun. 2020 Sep 23;11:4803. doi: 10.1038/s41467-020-18582-7 (PMC7511976; doi:10.1038/s41467-020-18582-7)
Supplement: Supplementary file 11 — Reporting Summary [file 41467_2020_18582_MOESM11_ESM.pdf]

nature research

Corresponding author(s): David R. Raleigh, MD, PhD

Last updated by author(s): Aug 25, 2020

Reporting Summary

Nature Research wishes to improve the reproducibility of the work that we publish. This form provides structure for consistency and transparency in reporting. For further information on Nature Research policies, see Authors & Referees and the Editorial Policy Checklist .

Statistics

For all statistical analyses, confirm that the following items are present in the figure legend, table legend, main text, or Methods section.

n/a	Confirmed	
☐	☒	The exact sample size (*n*) for each experimental group/condition, given as a discrete number and unit of measurement
☐	☒	A statement on whether measurements were taken from distinct samples or whether the same sample was measured repeatedly
☐	☒	The statistical test(s) used AND whether they are one- or two-sided<br>*Only common tests should be described solely by name; describe more complex techniques in the Methods section.*
☐	☒	A description of all covariates tested
☐	☒	A description of any assumptions or corrections, such as tests of normality and adjustment for multiple comparisons
☐	☒	A full description of the statistical parameters including central tendency (e.g. means) or other basic estimates (e.g. regression coefficient) AND variation (e.g. standard deviation) or associated estimates of uncertainty (e.g. confidence intervals)
☐	☒	For null hypothesis testing, the test statistic (e.g. *F*, *t*, *r*) with confidence intervals, effect sizes, degrees of freedom and *P* value noted<br>*Give P values as exact values whenever suitable.*
☒	☐	For Bayesian analysis, information on the choice of priors and Markov chain Monte Carlo settings
☐	☒	For hierarchical and complex designs, identification of the appropriate level for tests and full reporting of outcomes
☐	☒	Estimates of effect sizes (e.g. Cohen's *d*, Pearson's *r*), indicating how they were calculated

Our web collection on statistics for biologists contains articles on many of the points above.

Software and code

Policy information about availability of computer code

Data collection	This manuscript contains no custom code or mathematical algorithms. A full description of the open source code used to collect the data in this study (including HISAT2, DESeq2, R package, and FASTQC) including the version of each algorithm, is available in the Methods section.
Data analysis	This manuscript contains no custom code or mathematical algorithms. A full description of the open source code used to analyze the data in this study (including HISAT2, DESeq2, R package, and FASTQC) , including the version of each algorithm, is available in the Methods section.

For manuscripts utilizing custom algorithms or software that are central to the research but not yet described in published literature, software must be made available to editors/reviewers. We strongly encourage code deposition in a community repository (e.g. GitHub). See the Nature Research guidelines for submitting code & software for further information.

Data

Policy information about availability of data

All manuscripts must include a data availability statement. This statement should provide the following information, where applicable:

- Accession codes, unique identifiers, or web links for publicly available datasets
- A list of figures that have associated raw data
- A description of any restrictions on data availability

DNA methylation and RNA sequencing data that support the findings of this study have been deposited in the NCBI Gene Expression Omnibus (https://www.ncbi.nlm.nih.gov/geo/) under the following accession numbers: GSE151067 (DNA methylation) and RNA sequencing (RNA sequencing). Single cell RNA-seq raw data is available at the Sequence Read Archive (https://www.ncbi.nlm.nih.gov/sra) under project accession PRJNA660307. The remaining data are available within the Article, Supplementary Information, or available from the author upon request.

Field-specific reporting

Please select the one below that is the best fit for your research. If you are not sure, read the appropriate sections before making your selection.

☒ Life sciences ☐ Behavioural & social sciences ☐ Ecological, evolutionary & environmental sciences

For a reference copy of the document with all sections, see nature.com/documents/nr-reporting-summary-flat.pdf

Life sciences study design

All studies must disclose on these points even when the disclosure is negative.

Sample size	Patients presented for resection of meningioma who gave consent for tumor sampling for research were included in the study, which was approved by the institutional review board (10-01318 and 18-24633). No sample size calculation was performed, and all samples from patients meeting inclusion and exclusion criteria were included, but sample collection stopped once 3 meningiomas from each of the 3 WHO grades were obtained (representing the requirement of 3 biologic replicates described below).
Data exclusions	Exclusion criteria included meningioma skull base location, prior embolization, and unsuitable or unsafe tumor or patient characteristics for spatially-distinct sampling.
Replication	All experiments were performed with at least 3 biologic replicates. All attempts at replication were successful.
Randomization	This was a prospective non-randomized study of human tumor samples with no intervention and equal interrogation of all samples. Thus controlling for covariants is not relevant.
Blinding	Investigators were blinded to clinical information and molecular subgroup of samples during all data analysis.

Reporting for specific materials, systems and methods

We require information from authors about some types of materials, experimental systems and methods used in many studies. Here, indicate whether each material, system or method listed is relevant to your study. If you are not sure if a list item applies to your research, read the appropriate section before selecting a response.

Materials & experimental systems

n/a	Involved in the study	
☐	☒	Antibodies
☐	☒	Eukaryotic cell lines
☒	☐	Palaeontology
☒	☐	Animals and other organisms
☐	☒	Human research participants
☒	☐	Clinical data

Methods

n/a	Involved in the study	
☒	☐	ChIP-seq
☒	☐	Flow cytometry
☒	☐	MRI-based neuroimaging

Antibodies

Antibodies used	anti-FOXM1 (Abcam, ab207298; labeling validated using human colon and colon cancer tissues)<br>MIB1 (Ventana Medical Systems, Santa Clara, CA, 790-4286; rabbit antibody validated using human lymph node and tonsil)<br>anti-Ki-67 (Abcam, Cambridge, UK, ab15580; rabbit antibody validated by gene knockout in cells), Alexa Fluor secondary antibodies (Life Technologies, Carlsbad, CA, H3570; labeling validated using secondary-only and Fc controls). Lot numbers unknown.
Validation	anti-FOXM1 (Abcam, ab207298; labeling validated using human colon and colon cancer tissues)<br>MIB1 (Ventana Medical Systems, Santa Clara, CA, 790-4286; rabbit antibody validated using human lymph node and tonsil)<br>anti-Ki-67 (Abcam, Cambridge, UK, ab15580; rabbit antibody validated by gene knockout in cells), Alexa Fluor secondary antibodies (Life Technologies, Carlsbad, CA, H3570; labeling validated using secondary-only and Fc controls)

Eukaryotic cell lines

Policy information about cell lines

Cell line source(s)	Human meningioma cell lines were derived from spatially-distinct tumor samples included in this study. M10G cells were stably transduced with these lentiviral particles to generate M10GdCas9-KRAB cells. WTC11 and HEK293T cells were obtained form ATCC.

Authentication	DNA methylation profiling was performed on primary meningioma cell lines to confirm concordance to tumors of origin
Mycoplasma contamination	All lines tested negative for mycoplasma
Commonly misidentified lines (See ICLAC register)	No commonly misidentified cell lines were used in this study.

Human research participants

Policy information about studies involving human research participants

Population characteristics

Patients undergoing resection of meningioma of all ages, genders, past and current diagnosis and treatment categories were included consecutively. Covariates are summarized in Supplemental Table 1, and are recapitulated here:

Patients 13
Median age (range) 65 years (52-79 years)
Male:Female (ratio) 4:9 (1:2.3)
Asian:Caucasian (ratio) 2:11 (1:5.5)
Primary:Recurrent (ratio) 6:7 (1:1.2)
Median size (range) 17 cm3 (4.7-100 cm3)
Extent of resection
  Gross total 11 (85%)
  Near total 2 (15%)
WHO grade
  I 7 (54%)
  II (atypical) 3 (23%)
  III (anaplastic) 3 (23%)

Recruitment

Patients undergoing resection of meningioma who gave consent for research were included in this study. All patients who undergo craniotomy for tumor resection at UCSF sign consent to provide tissue for research. Thus, there was no self-selection bias or other biases that may influence or impact our results.

Ethics oversight

Study approved by the UCSF Institutional Review Board (10-01318 and 18-24633).

Note that full information on the approval of the study protocol must also be provided in the manuscript.
